# Machine Learning to Identify Metabolic Subtypes of Obesity: A Multi-Center Study

**DOI:** 10.3389/fendo.2021.713592

**Published:** 2021-07-14

**Authors:** Ziwei Lin, Wenhuan Feng, Yanjun Liu, Chiye Ma, Dooman Arefan, Donglei Zhou, Xiaoyun Cheng, Jiahui Yu, Long Gao, Lei Du, Hui You, Jiangfan Zhu, Dalong Zhu, Shandong Wu, Shen Qu

**Affiliations:** ^1^ Endocrinology and Metabolism Center, National Metabolic Management Center, Division of Metabolic Surgery for Obesity and Diabetes, Shanghai Tenth People’s Hospital, School of Medicine, Tongji University, Shanghai, China; ^2^ Department of Radiology, University of Pittsburgh, Pittsburgh, PA, United States; ^3^ Department of Endocrinology, Drum Tower Hospital Affiliated to Nanjing University Medical School, Nanjing, China; ^4^ The Center of Gastrointestinal and Minimally Invasive Surgery, Chengdu Third People’s Hospital, Southwest Jiaotong University, Chengdu, China; ^5^ Department of Bariatric and Metabolic Surgery, Shanghai East Hospital, Tongji University, Shanghai, China; ^6^ College of Computer, National University of Defense Technology, Changsha, China; ^7^ Department of Biomedical Informatics, Department of Bioengineering, Intelligent Systems Program, University of Pittsburgh, Pittsburgh, PA, United States

**Keywords:** obesity, metabolism, insulin, uric acid, machine learning, clustering

## Abstract

**Background and objective:**

Clinical characteristics of obesity are heterogenous, but current classification for diagnosis is simply based on BMI or metabolic healthiness. The purpose of this study was to use machine learning to explore a more precise classification of obesity subgroups towards informing individualized therapy.

**Subjects and Methods:**

In a multi-center study (n=2495), we used unsupervised machine learning to cluster patients with obesity from Shanghai Tenth People’s hospital (n=882, main cohort) based on three clinical variables (AUCs of glucose and of insulin during OGTT, and uric acid). Verification of the clustering was performed in three independent cohorts from external hospitals in China (n = 130, 137, and 289, respectively). Statistics of a healthy normal-weight cohort (n=1057) were measured as controls.

**Results:**

Machine learning revealed four stable metabolic different obese clusters on each cohort. Metabolic healthy obesity (MHO, 44% patients) was characterized by a relatively healthy-metabolic status with lowest incidents of comorbidities. Hypermetabolic obesity-hyperuricemia (HMO-U, 33% patients) was characterized by extremely high uric acid and a large increased incidence of hyperuricemia (adjusted odds ratio [AOR] 73.67 to MHO, 95%CI 35.46-153.06). Hypermetabolic obesity-hyperinsulinemia (HMO-I, 8% patients) was distinguished by overcompensated insulin secretion and a large increased incidence of polycystic ovary syndrome (AOR 14.44 to MHO, 95%CI 1.75-118.99). Hypometabolic obesity (LMO, 15% patients) was characterized by extremely high glucose, decompensated insulin secretion, and the worst glucolipid metabolism (diabetes: AOR 105.85 to MHO, 95%CI 42.00-266.74; metabolic syndrome: AOR 13.50 to MHO, 95%CI 7.34-24.83). The assignment of patients in the verification cohorts to the main model showed a mean accuracy of 0.941 in all clusters.

**Conclusion:**

Machine learning automatically identified four subtypes of obesity in terms of clinical characteristics on four independent patient cohorts. This proof-of-concept study provided evidence that precise diagnosis of obesity is feasible to potentially guide therapeutic planning and decisions for different subtypes of obesity.

**Clinical Trial Registration:**

www.ClinicalTrials.gov, NCT04282837.

## Introduction

The effects of weight loss treatments on patients with obesity vary greatly between cohorts/individuals. This may relate to the heterogeneity of the disease in terms of clinical presentation and pathogenesis (1–5). Conventional classification of obesity is mainly by a single dimension, e.g., body mass index (BMI) (6) or healthy/unhealthy metabolism (7). However, the coarse classification made by BMI inaccurately reflects the complexity and heterogeneity of obesity (6, 8). The metabolic healthy/unhealthy classification criteria are also controversial (7, 9, 10), where the patient distribution in the unhealthy group can vary substantially, ranging from 25% to 94% in reported studies (11). Towards precision treatment, a more refined metabolic classification of obesity phenotypes is highly demanded for a personalized diagnosis, aiming to identify patients at elevated risk of certain metabolic disorders or obesity comorbidities at the initial diagnostic visit. This kind of refined classification can provide a more precise diagnosis and enable more individualized preventive interventions and early treatments (12).

Artificial intelligence techniques have been quickly adopted in medicine. Data-driven machine learning modeling provides an intelligent method to mine up large and multi-dimensional data for refined classification and quantitative analysis. Applying machine learning to the obesity field is emerging but limited (13–16). Recent work shows encouraging preliminary evidences that some latent phenotypes of obesity could be revealed by machine learning (14–16). However, these obesity classification paradigms lack the consideration of an important clinical factor, i.e., metabolic abnormality, are limited to using data measured from specific devices that are not routinely available in clinical practice, or are short of external validation.

The purpose of this study was to develop a refined obesity classification criterion through an unsupervised machine learning approach in the setting of a multi-center study, where four independent study cohorts (a total of 1438 patients with obesity and 1057 normal-weight controls) were used for obesity classification and validation, using three common/key clinical variables representing a multi-dimensional characterization of obesity progression in terms of metabolism, hormone, inflammation, and oxidation (17).

## Material and Methods

### Study Design

**Figure 1:**
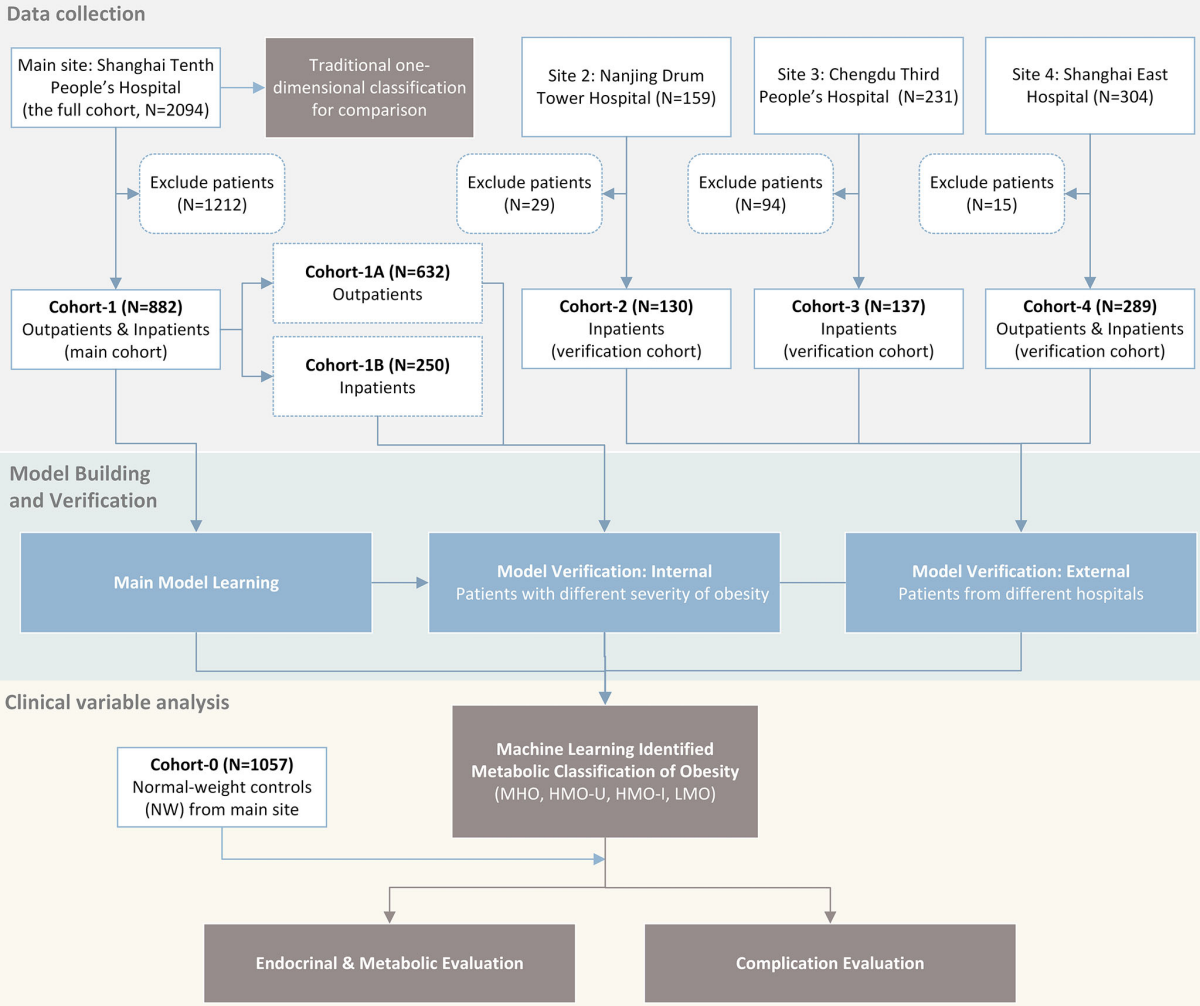
Flow chart of study design.

We conducted a multicenter study (ClinicalTrials No. NCT04282837) with approval from a local ethical committee and an institutional review board of the participating institutions. As shown in **Figure 1**, we retrospectively collected four patients cohorts (BMI ≥ 24kg/m^2^ according to the WHO criteria for overweight/obesity (6)) and one normal-weight control cohort from four different hospitals in P.R. China. We used one patient cohort (main cohort) for model learning and the rest three patient cohorts (verification cohorts) for verification. Detailed data analyses of the identified obesity subgroups were performed on the main cohort with a comparison to the control cohort.

### Study Sites and Population

Patients’ inclusion and exclusion criteria were shown in the **Supplemental Appendix**. From January 2010 to December 2019, at the Endocrinology and Metabolic Center of Shanghai Tenth People’s Hospital (main site), 2094 patients (the full cohort at the main site) with obesity were included, and 882 patients were included in Cohort-1 (main cohort) after exclusion (400 men and 482 women, median age 29 years, median BMI 35.9 kg/m^2^). Cohort-1 consisted of two sub-cohorts: Cohort-1A included 632 outpatients with obesity (296 men and 336 women, median age 28 years, median BMI 33.6 kg/m^2^). Most of these patients had relatively lower BMIs, mild to moderate obesity comorbidities, and were treated mainly with lifestyle interventions and weight-loss drugs. Cohort-1B included 250 inpatients with morbid obesity (104 men and 146 women, median age 31 years, median BMI 39.3 kg/m^2^) who were candidates for bariatric surgery according to the American Society of Metabolic and Bariatric Surgery/The Obesity Society/American Association of Clinical Endocrinologists guidelines (18) that are adjusted for Chinese patients. Pre-surgical examinations were performed before bariatric surgeries. Three follow-ups were conducted at 3, 6, and 12 months after surgery.

For the three verification cohorts, Cohort-2 included 130 patients with morbid obesity from Nanjing Drum Tower Hospital (60 men and 70 women, median age 30 years, median BMI 38.7 kg/m^2^). Cohort-3 included 137 patients with morbid obesity from Chengdu Third People’s Hospital (50 men and 87 women, median age 29 years, median BMI 38.0 kg/m^2^). Cohort-4 included 289 patients with moderate to morbid obesity from Shanghai East Hospital (81 men and 208 women, median age 30 years, median BMI 36.8 kg/m^2^). In addition, we collected a normal-weight healthy cohort (Cohort-0, n=1057) along with the main cohort from the main site, to use as controls (223 men and 834 women, median age 30 years, median BMI 21.2 kg/m^2^). These participants presented to clinics for routine health examinations. Key patient characteristics are shown in **Table 1**. Details on the measurement, calculation, and definition of endocrinological and metabolic disorders for each cohort were included in the **Supplemental Appendix**.

**Table 1:** Key characteristics of patients in each cohort.

	Cohort-0	Cohort-1A	Cohort-1B	Cohort-2	Cohort-3	Cohort-4
**N**	1057	632	250	130	137	289
**Man/Woman (woman %)**	223/834 (78.9%)	296/336 (53.2%)	104/146 (58.4%)	60/70 (53.8%)	50/87 (63.5%)	81/208 (72.0%)
**Age (years)**	30 (25, 36)	28 (21, 34)	31 (24, 36)	30 (23, 36)	29 (23, 35)	30 (25, 36)
**BMI (kg/m^2^)**	21.2 (19.9, 22.5)	33.6 (30.2, 37.5)	39.3 (35.7, 43.8)	38.7 (34.6, 43.1)	38.0 (34.5, 42.5)	36.8 (31.9, 42.5)
**Systolic pressure (mmHg)**	112 (106, 121)	133 (122, 145)	134 (125, 149)	137 (128, 150)	128 (124, 138)	NA
**Diastolic pressure (mmHg)**	70 (64, 77)	86 (78, 93)	81 (74, 90)	88 (79, 98)	83 (75, 96)	NA
**HbA1c (%)**	5.3 (5.0, 6.0)	5.7 (5.4, 6.3)	6.0 (5.6, 6.8)	5.8 (5.4, 6.4)	5.7 (5.4, 6.3)	5.8 (5.4, 6.3)
**Fasting glucose (mmol/l)**	4.6 (4.3, 5.0)	5.2 (4.8, 5.8)	5.5 (5.0, 6.8)	5.1 (4.7, 6.0)	5.4 (4.9, 6.4)	5.4 (5.0, 6.2)
**OGTT 2hr glucose (mmol/l)**	6.2 (5.1, 7.6)	7.4 (6.1, 9.4)	8.9 (6.7, 12.3)	8.0 (6.6, 10.1)	7.2 (5.9, 10.0)	NA
**Fasting insulin (mU/l)**	9.13 (5.68, 13.57)	25.45 (17.08, 35.48)	28.19 (19.42, 42.63)	26.66 (19.22, 38.49)	33.09 (20.85, 49.21)	27.87 (18.74, 42.24)
**OGTT 2hr insulin (mU/l)**	64.30 (39.98, 101.90)	125.95 (71.02, 217.00)	137.10 (66.10, 233.90)	139.30 (80.61, 221.50)	112.10 (68.28, 192.20)	NA
**Total cholesterol (mmol/l)**	4.22 (3.76, 4.67)	4.78 (4.14, 5.44)	4.53 (3.92, 5.19)	4.52 (3.96, 5.27)	5.08 (4.31, 5.59)	NA
**Triglyceride (mmol/l)**	0.74 (0.60, 1.01)	1.61 (1.15, 2.25)	1.53 (1.17, 2.15)	1.71 (1.15, 2.17)	1.76 (1.29, 2.57)	NA
**Uric acid (μmol/l)**	267 (236, 303)	412 (341, 481)	412 (359, 481)	464 (373, 533)	439 (377, 518)	391 (318, 454)
**Creatinine (μmol/L)**	56.0 (50.0, 62.0)	68.1 (57.0, 77.8)	59.4 (53.4, 68.7)	55.5 (47.0, 66.0)	60.9 (49.0, 68.3)	NA
**ALT (U/L)**	13 (10, 16)	43 (24, 75)	42 (25, 77)	46 (28, 80)	47 (24, 81)	NA
**AST (U/L)**	18 (15, 22)	28 (20, 43)	27 (18, 43)	30 (20, 46)	29 (20, 53)	NA

Values are shown as median (IQR 25-75%). ALT, alanine aminotransferase; AST, aspartate aminotransferase; BMI, body mass index; HbA1c, glycosylated hemoglobin a1c; NA, not available; OGTT, oral glucose tolerance test.

### Key Clinical Variables Selection for Machine Learning

Based on the consensus of our study team consisting of multiple expert physicians in obesity/endocrinology, the clinical variables we used to build classification models should be those related to metabolism, hormones, inflammation, and antioxidation, which represent the underlying progression mechanisms of obesity comorbidities. We selected key clinical variables out of hundreds of metabolic parameters based on the following criteria: (i) essential to characterize obesity, (ii) routinely acquired/measured in clinics, and (iii) easy to interpret with a physical meaning. We also intended to select a small number of variables to improve the generalizability of the classification models. Based on these criteria, we performed a data-driven experiment to select potential variables and optimal model parameters for the classification.

### Unsupervised Modeling by Clustering Algorithms

We used and compared two clustering algorithms [i.e., k-means (19) and two-step (20)] for machine learning. Clustering was implemented by SPSS Modeler version 18 (IBM, Chicago, USA). All variables were normalized (mean value of 0 and standard deviation [SD] of 1) before the cluster analysis. The k-means clustering was implemented with different k values (maximum iterations of 30 and change tolerance of 0.00001) and the one with minimum silhouette widths was used in the end. In the two-step clustering, the first step estimates the optimal number of clusters on the basis of silhouette width and the second step performs hierarchical clustering using log-likelihood as a distance measure and Schwarz’s Bayesian criterion for clustering. Considering the notable differences of key patient characteristics induced by patient sex, we built a model with two sub-models that were separately trained on female and male patients, and then the classification results of the sub-models were pooled for further analysis.

### Clustering on Main Cohort and Verification on Three Other Cohorts

Clustering algorithms were first applied to the main cohort, Cohort-1, and the resulting clusters were used as the main classification model. Verification was performed by applying the same clustering algorithms to Cohort-1A, Cohort-1B, Cohort-2, Cohort-3, and Cohort-4, separately, and the resulting clusters of each verification cohort were compared to the clusters of the main model in terms of patients’ distribution percentages and characteristics in each cluster. The denotation of clusters was assigned referring to the characteristics of the three classification variables. In order to further measure the generalizability of the classification models, we also assigned patients in each verification cohort to the clusters derived from the main model, according to the similarity of a patient’s characteristics to each of the clusters in the main model. The similarity was calculated as their Euclidian distance (for k-means clustering) or log-likelihood distance (for two-step clustering) from the nearest cluster center derived from the main model. Then sensitivity, specificity, and accuracy for clustering, as well as the inter-cluster Jaccard coefficients (21), were calculated.

### Missing Value Imputation

The area under the curve (AUC) of glucose (glucose _AUC_) and insulin (insulin _AUC_) were calculated using the trapezoidal rule at four data points of 0, 30, 60, and 120 min during oral glucose tolerance test (OGTT) for patients in Cohort-1 and Cohort-2. However, since the four-time-points OGTT was not a routine measurement for patients in Cohort-3 and Cohort-4 (which is not uncommon in certain hospitals), certain time-points of the OGTT data were missing for these patients. Thus, we built a group of linear regression models using the complete OGTT data available in Cohort-1, and employed one to three time-points of OGTT to estimate the four-time-points glucose _AUC_ and insulin _AUC_. The linear regression models for the estimation of glucose _AUC_ and insulin _AUC_ were trained and tested in 70% and 30%, respectively, of the data from Cohort-1 using stepwise method. The F test was performed with P < 0.05 for inclusion and P > 0.1 for exclusion, and outlier tolerance of 0.0001. The estimates of the glucose _AUC_ and insulin _AUC_ showed an average adjusted R in the test set of 0.954 (range: 0.860-0.996) for glucose _AUC_ and 0.873 (range: 0.643-0.984) for insulin _AUC_ (**Tables S1** and **S2**).

### Statistical Analysis

Clinical implications of variables related to metabolism and morbidity were compared with respect to the four obesity clusters. Continuous variables were expressed as the median (interquartile range 25-75%), since most of them were a skewed distribution. Variables not normally distributed were logarithmically or square root transformed before statistical analysis, which was performed with SPSS version 26 (IBM, Chicago, USA). Differences for continuous variables were assessed by performing ANOVA or ANCOVA, as appropriate. Bonferroni correction was used for the *post hoc* analysis. Differences in ratio variables were assessed by Chi-square test. To identify the odds of obesity comorbidities in different subgroups, a binary logistic regression analysis was performed, and odd ratio (OR) or OR adjusted (AOR) for sex and age and the corresponding 95% confidence interval (CI) were calculated. For all analyses, *p* values were two-tailed and *p* < 0.05 was considered statistically significant.

## Results

### Baseline Obesity Classification

Of the 2094 patients (i.e., the full cohort at the main site), 300 (14%) and 1794 (86%) were overweight and obese, respectively, in terms of BMI. The proportions of metabolic unhealthy patients varied substantially, where 36-93% overweight and 55-97% obese patients were observed according to different criteria (**Figure S1A–D**). In the BMI-based categorization, while the incidence of several metabolic diseases (e.g., hypertension, metabolic syndromes, and hyperuricemia) increased gently along with the BMI categories, there were no obvious differences in the clinical characteristics among the four BMI subgroups (**Figure S1E–F**).

### Obesity Subtypes Identified by Machine Learning/Clustering

**Figure 2:**
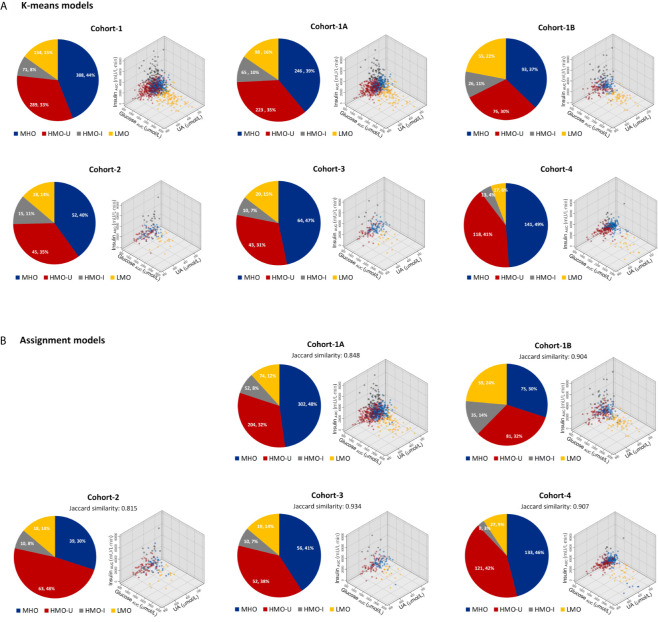
Patient distributions and characteristics in each cohort with respect to the four clusters generated from machine learning. **(A)** Clusters generated independently from each individual cohort by using k-means. **(B)** Clusters generated by assigning patients in each verification cohort to the main model generated from Cohort-1. Data in the pie plots were shown as N (patient number) and its percentage over the full cohort. HMO-I, hypermetabolic obesity hyperinsulinemia subtype; HMO-U, hypermetabolic obesity hyperuricemia subtype; LMO, hypometabolic obesity; MHO, metabolic healthy obesity.

Data-driven experiments selected the following three variables as key clustering factors: 1) glucose _AUC_, reflecting the severity of disturbances in energy metabolism; 2) insulin _AUC_, reflecting the compensatory balance of hormones to the increased somatogenic need; and 3) uric acid (UA), reflecting the inflammation and oxidation in the body. In Cohort-1 (the main cohort), k-means yielded four distinct clusters with minimum silhouette widths (**Figure 2A**). The two-step clustering showed similar clustering results as k-means (Jaccard similarity 0.831, **Figure S2A**). The cluster centers were shown in **Table 2** and **Table S3**. Here we report the results on k-means only (see **Supplemental Appendix** for two-step results).

**Table 2:** Cluster centers in Cohort-1 with k-means method.

	MHO	HMO-U	HMO-I	LMO
**Men**				
** Glucose _AUC_, mmol/L·min**	993	982	1008	1845
** Insulin _AUC_, mU/L·min**	17089	19951	50543	9477
** Uric acid, μmol/L**	405	585	489	404
**Women**				
** Glucose _AUC_, mmol/L·min**	928	1100	1094	1829
** Insulin _AUC_, mU/L·min**	12894	21895	54645	9320
** Uric acid, μmol/L**	331	454	392	356

HMO-U, hypermetabolic obesity-hyperuricemia subtype; HMO-I, hypermetabolic obesity-hyperinsulinemia subtype; LMO, hypometabolic obesity; MHO, metabolic healthy obesity; AUC, area under the curve during oral glucose tolerance test.

**Figure 3:**
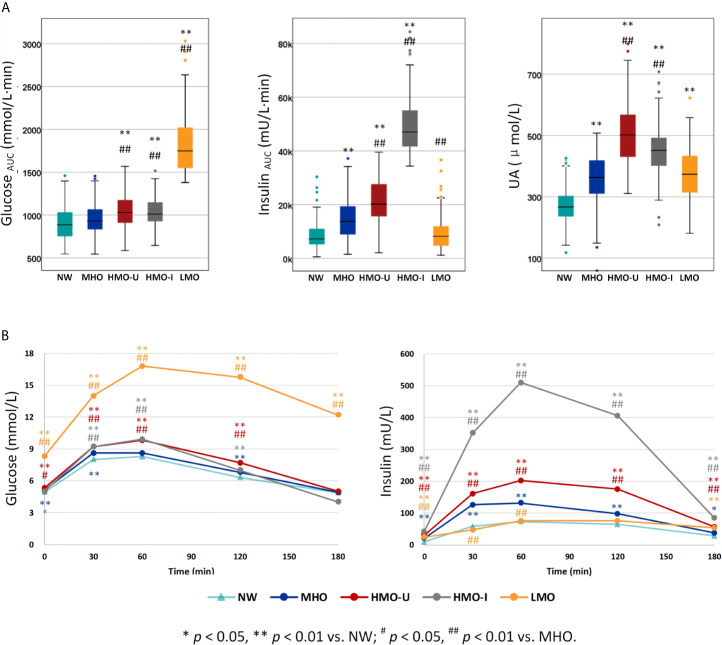
Comparison of the three classification variables across the four clusters generated from Cohort-1 using k-means. **(A)** The three classification variables. **(B)** Oral glucose tolerance test (OGTT) curves for glucose and insulin. *P* values refer to results after adjustment for age and sex. HMO-I, hypermetabolic obesity hyperinsulinemia subtype; HMO-U, hypermetabolic obesity hyperuricemia subtype; LMO, hypometabolic obesity; MHO, metabolic healthy obesity; NW, normal-weight control; UA, uric acid.

The four clusters that resulted from Cohort-1 were as follows (**Figure 3A**): Cluster 1 (denoted as metabolic healthy obesity [MHO]): 388 (44%) patients characterized by relatively healthy-metabolic statues, with normal glucose (median glucose _AUC_: 928 vs. 886 mmol/l·min in normal-weight), slight compensated insulin secretion (median insulin _AUC_: 13775 vs. 7252 mU/l·min in normal-weight), and mild increased UA (median: 363 vs. 267 μmol/l in normal-weight). Cluster 2 (denoted as hypermetabolic obesity hyperuricemia subtype [HMO-U]) and cluster 3 (denoted as hypermetabolic obesity hyperinsulinemia subtype [HMO-I]): included 289 (33%) and 71 (8%) patients, respectively, both characterized by slightly increased glucose, compensated insulin secretion, and increased UA. HMO-U was distinguished by high UA (median: 501 vs. 363-451 μmol/l in other three subgroups), whereas HMO-I was distinguished by overcompensated insulin secretion (median insulin _AUC_: 47061 vs. 8244-20186 mU/l·min in other three subgroups). Cluster 4 (denoted as hypometabolic obesity [LMO]): 134 (15%) patients characterized by high glucose (median glucose _AUC_: 1748 vs. 928-1030 mmol/l·min in other three subgroups) with decompensated insulin secretion (median insulin _AUC_: 8244 vs. 13775-47061 mU/l·min in the other three subgroups). The characteristics of the four clusters were similar between the clusters generated by male and female patients, separately (**Figure S3**). HMO-U, HMO-I, and LMO can be grouped with a single notion of metabolic unhealthy obesity (MUO) in comparison to MHO. **Figure 3B** showed the glucose and insulin curves during OGTT across the four clusters.

### Verification of the Obesity Subtyping on Other Cohorts

In each verification cohort, there were also four distinct clusters generated with similar patient distributions and characteristics to the corresponding clusters of the main model (**Figure 2A**). In Cohort-4, the proportions of patients clustered into LMO and HMO-I were relatively small, which may potentially have to do with the estimation of missing values for glucose _AUC_ and insulin _AUC_, respectively.

As shown in **Figure 2B** and **Table 3**, in terms of the assignment of patients into the clusters derived from the main model, the mean assignment accuracy was 0.941, ranging from 0.908 to 0.967; the mean Jaccard similarity coefficient was 0.882, ranging from 0.815 to 0.934 for assignment-generated clusters vs. independent k-means-generated clusters, except for two notable and reasonable differences as explained in the following: (i) A higher proportion of patients were assigned to MUO in Cohort-1B in comparison to Cohort-1A, which actually reflects the difference of patients’ severity of obesity in the two sub-cohorts. (ii) A higher proportion of patients were assigned to HMO-U in Cohort-2, which reflects the higher average UA in this cohort (**Table 1**). Similar results were also observed with the two-step clustering methods (**Figure S2**, **Table S4**).

**Table 3:** Performance of assigning patients in each verification cohort to the four clusters generated by k-means on the main cohort, Cohort-1.

	Cohort-1A			Cohort-1B			Cohort-2			Cohort-3			Cohort-4			Mean		
	ACC	SEN	SPE	ACC	SEN	SPE	ACC	SEN	SPE	ACC	SEN	SPE	ACC	SEN	SPE	ACC	SEN	SPE
**MHO**	0.905	0.992	0.850	0.948	0.892	0.981	0.854	0.692	0.962	0.942	0.875	1.000	0.924	0.894	0.953	0.914	0.869	0.949
**HMO-U**	0.875	0.780	0.927	0.908	0.908	0.908	0.831	0.956	0.765	0.934	1.000	0.904	0.941	0.941	0.942	0.898	0.917	0.889
**HMO-I**	0.954	0.677	0.986	0.972	0.731	1.000	0.962	0.667	1.000	1.000	1.000	1.000	0.983	0.615	1.000	0.974	0.738	0.997
**LMO**	0.962	0.755	1.000	0.980	1.000	0.974	0.985	0.944	0.991	0.993	0.950	1.000	0.965	1.000	0.963	0.977	0.930	0.986
**Mean**	0.924	0.801	0.941	0.952	0.883	0.966	0.908	0.815	0.929	0.967	0.956	0.976	0.953	0.862	0.964	0.941	0.863	0.955

ACC, Accuracy; HMO-I, hypermetabolic obesity hyperinsulinemia subtype; HMO-U, hypermetabolic obesity hyperuricemia subtype; LMO, hypometabolic obesity; MHO, metabolic healthy obesity; SEN, Sensitivity; SPE, Specificity.

### Metabolic Feature Analysis With Respect to the Four Subtypes

As shown in **Table 4**, HMO-I contained more male patients (67.6 vs. 41.2-48.5% in other three subgroups) and had the lowest age at onset of obesity (median: 13 vs. 16-20 years in other three subgroups), while LMO had the longest duration of obesity at visit (median: 12 vs. 8-9 years in other three subgroups). Patients in LMO presented the most severe central obesity with the highest percentage of fat mass deposited at the trunk (median trunk/limb fat mass ratio: 1.41 vs. 1.18-1.26 in other three subgroups; median trunk/leg fat percentage ratio: 1.24 vs. 1.10-1.18 in other three subgroups). Patients in HMO-I had the highest incidence of acanthosis nigricans (74.6 vs. 36.9-59.1% in other three subgroups).

**Table 4:** Comparison of clinical variables across the four clusters generated from Cohort-1 using k-means and from the normal-weight controls.

	NW	MHO	HMO-U	HMO-I	LMO
**N**	1057	388	289	71	134
**Basic demographic information and obesity history**					
** Female patients, %**	78.9% ^§§^	56.7%	58.8%	32.4% ^§§^	51.5%
** Age, years**	30 (25, 36)	29 (23, 35)	27 (19, 33) ^**##^	25 (18, 31) ^** ##^	32 (28, 39) ^** ##^
** Age at onset of obesity, years**	NA	20 (10, 27)	16 (12, 25)	13 (7, 17) ^#^	19 (7, 27)
** Duration of obesity, years**	NA	8 (4, 16)	8 (5, 15)	9 (4, 14)	12 (7, 22) ^##^
**Anthropometric examinations and fat distribution**					
** BMI, kg/m^2^**	21.2 (19.9, 22.5)	33.8 (30.2, 37.3)	36.6 (32.9, 40.4) ^** ##^	36.4 (33.2, 40.3) ^** ##^	35.3 (31.2, 40.2) ^** ##^
** Excess weight/healthy weight, %**	NA	40.7 (25.7, 55.5)	52.5 (37.2, 68.4) ^##^	51.6 (38.4, 67.8) ^#^	47.1 (29.8, 64.4) ^#^
** Waist to hip ratio**	NA	0.95 (0.90, 1.00)	0.96 (0.92, 1.01) ^##^	0.99 (0.95, 1.03) ^#^	1.00 (0.96, 1.05) ^#^
** Trunk/limb fat mass ratio (DXA)**	NA	1.26 (1.08, 1.41)	1.22 (1.08, 1.39)	1.18 (1.10, 1.33)	1.41 (1.25, 1.67) ^#^
** Trunk/legs fat percentage ratio (DXA)**	NA	1.10 (1.06, 1.23)	1.12 (1.03, 1.19)	1.18 (1.07, 1.28)	1.24 (1.13, 1.36) ^#^
** Patients with acanthosis nigricans, %**	NA	36.9%	59.1% ^##^	74.6% ^##^	39.7%
**Glucose metabolism**					
** HbA1c, %**	5.3 (5.0, 6.0)	5.6 (5.4, 6.1) ^**^	5.7 (5.4, 6.2) ^**^	5.7 (5.4, 5.8)	8.0 (6.8, 9.3) ^** ##^
** HOMA-β**	124 (78, 193)	263 (176, 407) ^**^	336 (238, 460) ^** ##^	584 (474, 962) ^** ##^	97 (57, 166) ^* ##^
** IGI**	16.58 (9.68, 26.32)	28.97 (18.43, 43.30) ^**^	33.69 (20.07, 51.90) ^**^	76.48 (51.39, 111.61) ^** ##^	4.72 (2.18, 8.50) ^** ##^
** HOMA-IR**	2.01 (1.20, 3.06)	4.78 (3.33, 7.17) ^**^	7.20 (5.21, 9.79) ^** ##^	9.67 (7.76, 13.19) ^** ##^	9.45 (6.01, 13.92) ^** ##^
** WBISI**	4.25 (2.90, 6.28)	2.01 (1.40, 2.90) ^**^	1.27 (1.00, 1.65) ^** ##^	0.74 (0.55, 0.86) ^** ##^	1.26 (0.92, 2.32) ^** ##^
** DI (HOMA-β/HOMA-IR)**	63.83 (44.97, 89.44)	55.15 (40.91, 72.12) ^**^	47.17 (33.73, 65.60) ^** ##^	60.00 (43.86, 88.93)	11.30 (6.75, 17.61) ^** ##^
** DI (IGI × WBISI)**	71.91 (39.81, 116.90)	58.06 (33.30, 94.62) ^**^	41.60 (23.71, 67.77) ^** ##^	58.93 (34.37, 80.13) ^**^	6.64 (3.10, 11.69) ^** ##^
**Lipid metabolism**					
** Total cholesterol, mmol/L**	4.22 (3.76, 4.67)	4.53 (4.03, 5.18) ^**^	4.73 (4.20, 5.52) ^** ##^	4.84 (4.20, 5.41) ^**^	4.91 (4.17, 5.51) ^**^
** LDL-c, mmol/L**	2.30 (2.00, 2.76)	2.76 (2.31, 3.34) ^**^	2.97 (2.52, 3.60) ^** ##^	3.05 (2.61, 3.47) ^**^	2.98 (2.14, 3.59) ^**^
** HDL-c, mmol/L**	1.30 (1.20, 1.57)	1.08 (0.95, 1.24) ^**^	1.02 (0.90, 1.16) ^**^	0.96 (0.85, 1.08) ^**^	1.01 (0.84, 1.13) ^** #^
** Triglyceride, mmol/L**	0.74 (0.60, 1.01)	1.39 (1.02, 2.00) ^**^	1.59 (1.20, 2.22) ^**^	1.66 (1.25, 2.12) ^**^	1.99 (1.56, 2.86) ^** ##^
** Patients with carotid plaque or increased IMT (ultrasound), %**	NA	10.2%	15.6%	18.2%	38.7% ^##^
**Gynecological diseases**					
** Testosterone (women), nmol/L**	NA	0.96 (0.57, 1.38)	1.50 (0.90, 2.09) ^##^	1.72 (0.79, 2.24)	1.18 (0.67, 1.92)
** Patients with PCO (ultralsound), %**	NA	14.5%	19.7%	66.7% ^##^	10.0%

P values after Bonferroni correction are adjusted for age and sex except the analysis of basic demographic information and obesity history. BMI, body mass index; DI, Disposition indices; DXA, Dual energy x-ray absorptiometry; HbA1c, glycosylated hemoglobin a1c; HDL-c, high density lipoprotein cholesterol; HMO-I, hypermetabolic obesity hyperinsulinemia subtype; HMO-U, hypermetabolic obesity hyperuricemia subtype; HOMA-IR, homeostatic model assessment of insulin resistance; HOMA-β, homoeostasis model assessment of β-cell function; IGI, insulinogenic index; IMT, intima-media thickness; LDL-c, low density lipoprotein cholesterol; LMO, hypometabolic obesity; MHO, metabolic healthy obesity; NA, not applicable / not available; NW, normal-weight control; PCO, polycystic ovaries; WBISI, whole-body insulin sensitivity index. ^ *^P< 0.05, ^**^P < 0.01 vs. NW; ^#^P < 0.05, ^##^P < 0.01 vs. MHO; ^§§^p < 0.01 vs. the whole obesity cohort distribution (only for the analysis of sex distribution)

As expected, patients in MHO showed a relatively healthy endocrinal and metabolic status in the four subgroups of obesity. Patients in HMO-I showed the worst hepatic and peripheral insulin sensitivity (median whole-body insulin sensitivity index [WBISI]: 0.74 vs. 1.26-2.01 in other three subgroups) and overcompensated insulin secretion (median insulinogenic index [IGI]: 76.5 vs. 4.7-33.7 in other three subgroups; median homoeostasis model assessment of β-cell [HOMA-β]: 583 vs. 97-336 in other three subgroups), which resulted in a balance and made the disposition index of glucose (DI) and glycosylated hemoglobin a1c (HbA1c) similar to MHO or even to normal-weight (median DI [IGI×WBISI]: 58.9 vs. 58.06 and 71.9 in MHO and normal-weight, respectively; median DI [HOMA-β/HOMA-IR]: 60.0 vs. 55.1 and 63.8 in MHO and normal-weight, respectively; median HbA1c: 5.7 vs. 5.6 and 5.3% in MHO and normal-weight, respectively). Patients in LMO also showed severe hepatic insulin resistance (median homeostatic model assessment of insulin resistance [HOMA-IR]: 9.45 vs. 4.78-9.67 in other three subgroups) but decompensated insulin secretion (median IGI: 4.7 vs. 28.9-76.5 in other three subgroups; median HOMA-β: 97 vs. 262-583 in other three subgroups), which resulted in the significantly decreased disposition ability of glucose (median DI [IGI×WBISI]: 6.6 vs. 41.6-58.9 in other three subgroups; median DI [HOMA-β/HOMA-IR]: 11.3 vs. 47.1-60.0 in other three subgroups) and increased HbA1c (median: 8.0 vs. 5.6-5.7% in other three subgroups). Meanwhile, LMO showed the most severe lipid metabolism (median triglyceride [TG]: 1.99 vs. 1.39-1.66 in other three subgroups of obesity), together with the highest incidence of carotid plaque and increased intima-media thickness (IMT) (38.7 vs. 10.2-18.2% in other three subgroups). In the examination of gonad disorders in women, patients in HMO-I showed a greater increased incidence of polycystic ovaries (PCO) examined by ultrasound (66.7 vs. 10.0-19.7% in other three subgroups). See **Tables S5, S6** for more comparisons of data.

### Comorbidity Analysis With Respect to the Four Subtypes

**Figure 4:**
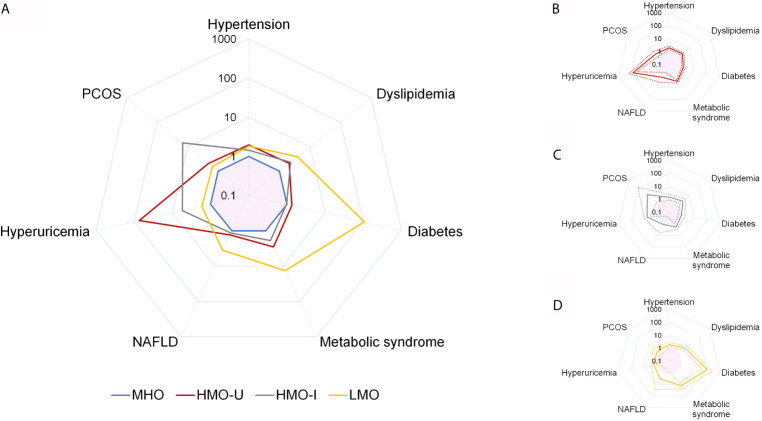
Comparison of adjusted odds ratio (AOR) for obesity comorbidities across the four clusters generated from Cohort-1 using k-means. Comparison of AOR across the four clusters **(A)**, and AOR and 95%CI for HMO-U **(B)**, HMO-I **(C)**, and LMO **(D)**. MHO is used as the reference category. Analyses are adjusted for sex and age. Values are shown as AOR (solid line) and/or 95%CI (dash line). Pink shadow in the center of each spider diagram is area for AOR < 1. HMO-I, hypermetabolic obesity hyperinsulinemia subtype; HMO-U, hypermetabolic obesity hyperuricemia subtype; LMO, hypometabolic obesity; MHO, metabolic healthy obesity; NAFLD, nonalcoholic fatty liver disease; PCOS, polycystic ovary syndrome.

Comorbidity analyses were shown in **Figure 4** and **Table 5**. Consistent with the metabolic examinations described above, patients in MHO showed a relatively low risk of comorbidities in the four subgroups of obesity. Patients in HMO-U and HMO-I showed a slightly increased risk of metabolic diseases compared to MHO, except for a significant increased risk of hyperuricemia (AOR 73.67 to MHO, 95%CI 35.46-153.06) and polycystic ovary syndrome (PCOS) (AOR 14.44 to MHO, 95%CI 1.75-118.99), respectively. Patients in LMO showed the worst metabolism with the highest risk of diabetes (AOR 105.85 to MHO, 95%CI 42.00-266.74) and metabolic syndrome (AOR 13.50 to MHO, 95%CI 7.34-24.83). The prognosis analyses of bariatric surgery patients were shown in **Figure S4**. The summary of the clinical characteristics and suggested treatments with respect to the four subgroups were shown in **Table 6**.

**Table 5:** The adjusted odds ratio (AOR) of comorbidities across the four clusters generated from Cohort-1 using k-means.

	MHO	HMO-U	HMO-I	LMO
**Hypertension**	1.00	1.97 (1.44-2.69) ^##^	1.44 (0.86-2.41)	1.85 (1.22-2.80) ^##^
**Diabetes**	1.00	1.34 (0.96-1.87)	0.98 (0.54-1.79)	105.85 (42.00-266.74) ^##^
**Dyslipidemia**	1.00	2.08 (1.51-2.87) ^##^	2.32 (1.31-4.11) ^##^	3.88 (2.29-6.57) ^##^
**Hypercholesterolemia**	1.00	1.61 (1.16-2.22) ^##^	1.63 (0.96-2.77) ^#^	1.70 (1.14-2.55) ^#^
**Hypertriglyceridemia**	1.00	1.79 (1.33-2.43) ^##^	1.73 (1.05-2.84) ^#^	2.92 (1.96-4.36) ^##^
**Low-HDL**	1.00	1.60 (1.17-2.19) ^##^	2.26 (1.37-3.72) ^##^	2.16 (1.45-3.20) ^##^
**High-LDL**	1.00	1.88 (1.35-2.61) ^##^	1.91 (1.13-3.24) ^#^	1.55 (1.02-2.35) ^#^
**Metabolic syndrome**	1.00	2.86 (2.09-3.92) ^##^	1.89 (1.14-3.14) ^#^	13.50 (7.34-24.83) ^##^
**Hyperuricemia**	1.00	73.67 (35.46-153.06) ^##^	5.48 (3.08-9.77) ^##^	1.70 (1.15-2.50) ^##^
**Women hyper-testosterone**	1.00	3.38 (2.15-5.32) ^##^	2.85 (1.17-6.93) ^#^	2.29 (1.22-4.31) ^#^
**PCOS**	1.00	2.07 (1.08-3.95) ^#^	14.44 (1.75-118.99) ^#^	1.53 (0.57-4.14)
**Men hypo-testosterone**	1.00	2.06 (1.29-3.28) ^##^	2.99 (1.53-5.82) ^##^	2.19 (1.20-3.97) ^#^
**Microalbuminuria**	1.00	2.68 (1.2-5.95) ^#^	0.47 (0.09-2.39)	3.35 (1.41-7.98) ^##^
**NAFLD**	1.00	1.30 (0.48-3.49)	1.15 (0.22-5.96)	3.53 (0.43-29.17)
**NAFLD with elevated ALT, AST**	1.00	1.97 (1.15-3.38) ^#^	1.47 (0.66-3.30)	1.98 (1.01-3.85) ^#^

Values are shown as AOR (95%CI). Analyses are adjusted for sex and age. ALT, alanine aminotransferase; AST, aspartate aminotransferase; HDL, high density lipoprotein cholesterol; HMO-I, hypermetabolic obesity hyperinsulinemia subtype; HMO-U, hypermetabolic obesity hyperuricemia subtype; LDL, low density lipoprotein cholesterol; LMO, hypometabolic obesity; MHO, metabolic healthy obesity; NAFLD, nonalcoholic fatty liver disease; PCOS, polycystic ovary syndrome. MHO is the reference category. ^#^P < 0.05, ^##^P < 0.01 vs. MHO.

**Table 6:** Summarization of the patient characteristics in the four obesity subtypes.

	MHO	HMO-U	HMO-I	LMO
**Percentage in obesity**	44%	33%	8%	15%
**Sex**	Equal	Equal	More men	Equal
**Age at onset and duration of obesity**	Average	Average	Youngest age at onset	Longest duration at visit
**Acanthosis nigricans**	The lowest prevalence in obesity	Higher prevalence compared with MHO	The highest prevalence in obesity	Similar to MHO
**Fat distribution**	Relative lower BMI compared with the other 3 subgroups	Higher BMI compared with MHO	Higher BMI compared with MHO	Higher BMI compared with MHO, with the most severe central obesity
**Glucometabolism**	Normal glucose with slightly compensated insulin secretion	Slightly increased glucose with moderately compensated insulin secretion	Slightly increased glucose with overcompensated insulin secretion	The highest glucose with decompensated insulin secretion, and the highest prevalence of diabetes in obesity
**Insulin resistance**	Slightly insulin resistance	Moderate insulin resistance	The most severe liver and peripheral insulin resistance	Severe liver insulin resistance
**Lipid profile**	Worse compared with NW	Worse than MHO	Worse than MHO	The worst in obesity with the most severe carotid lipid deposition
**Uric acid**	Slightly increased compared with NW	The highest in obesity	Moderately increased compared with MHO	Similar to MHO
**PCOS**	Relative lower in obesity	Increased testosterone (women) and incidence of PCOS compared with MHO	Largely increased incidences of PCO and PCOS compared with MHO	Similar to MHO
**Potential treatment suggestions**	Weight loss	Weight loss	Weight loss	Weight loss
	Symptom therapy if it’s necessary	Focus on uric acid management but avoid overtreatment	Focus on the relief of hyperinsulinemia Appropriate hormonotherapy if it’s necessary	Focus on the management of glucolipid metabolism and restoration of pancreatic β-cell function

BMI, body mass index; HMO-I, hypermetabolic obesity hyperinsulinemia subtype; HMO-U, hypermetabolic obesity hyperuricemia subtype; LMO, hypometabolic obesity; MHO, metabolic healthy obesity; NW, normal-weight control; PCO, polycystic ovary; PCOS, polycystic ovary syndrome.

## Discussion

We leveraged machine learning to identify a refined classification of patients with obesity from multiple hospitals. The classification yielded four metabolically distinct clusters (i.e., MHO, HMO-U, HMO-I, and LMO), which showed a high degree of agreement/reproducibility among the four independent cohorts. This multi-dimensional classification provides an enhanced capacity over traditional healthy/unhealthy obesity and BMI categorizations to reflect the complexity and heterogeneity of metabolic disorders, thereby having the potential to enable more precise preventions, diagnoses, and therapy planning. To the best of our knowledge, this is the first study to apply unsupervised machine learning to common clinical variables to refine metabolic classification of obesity in a multi-center setting.

The algorithms for unsupervised clustering of data are critical for a machine learning study. We used independently two mature algorithms, i.e., k-means and two-step, and observed highly similar classification results, indicating our data clustering is relatively robust. The assignment of patients in the verification cohorts to the main model led to high Jaccard similarity coefficients (range 0.815-0.934), indicating our classification effects are stable when tested on the multiple cohorts Jaccard similarity of greater than 0.750 is considered as a stable clustering (21).

We used three common clinical variables for classification. While this is a relatively large granularity for clustering, the three variables reflect important dimensions (i.e., metabolism, hormone, as well as inflammation and oxidation) in characterizing obesity progression and are critical in providing important interpretation of etiopathogenesis to guide therapies. Four obesity subgroups were yielded from the three variables, and our analyses have revealed clinical insights associated with each subgroup to help us better understand obesity and guide clinical treatment planning. If more clinical variables are used for classification, more refined clustering may be identified. While that we emphasize the importance of applicability and generalizability of an obesity classification model - more variables for classification could lead to overfitting and may reduce applicability on patients without complete clinical variables. This study using three variables showed promising generalizability and, in future work, further evaluation on a different number of clinical variables on substantially larger cohorts are warranted. In addition, our study used clinically routinely acquired variables for classifications, which can enable a broader utility of such classification models. It was different from previous studies that used lifestyle data (14, 16) or data acquired using specific research devices (e.g., hypothalamic blood flow, gastric empty rate, and energy expenditure) (15). The limited body of previous work (14–16) also did not consider the important information on metabolic abnormality.

The four subgroups in our study have important implications on treatments. MHO showed a relative healthy metabolic statues and hormone balance, where patients should be motivated to achieve a normal weight for long-term considerations, as risks of metabolic disorders are still higher than normal-weight subjects and may increase over time (22). In contrast, LMO showed the most severe central obesity due to the severe hepatic insulin resistance (23), decompensated insulin secretion, and resultant poor metabolism (diabetes, dyslipidemia, and carotid lipid deposition). These patients may be more vulnerable to atherosclerosis and cardiometabolic diseases (24). Suggested treatments may include management of glucolipid metabolism and restoration of pancreatic β-cell function. HMO-U showed the worst UA metabolism but still relatively healthy glucolipid metabolism. For these patients, UA regulation may be an effective therapy, but note that overtreatment may attenuate the benefits of antioxidation by UA (25). HMO-I showed the worst hepatic and peripheral insulin sensitivity but overcompensated insulin secretion, which to some extent balanced the glucolipid metabolism. Severe insulin resistance may have resulted in the highest incidence of acanthosis nigricans (26) and PCOS (27). Therapies may be directed to relieve hyperinsulinemia for these patients.

Our study has some limitations. First, all patients are of Chinese in China. The applicability of our classification models to patients of other ethnicities requires further evaluation. Second, since this is a multi-center retrospective study, there may be noticeable differences in measurements and lab tests across different institutions. Third, the data imputation for patients with missing OGTT time point data may have introduced inaccurate estimates, while consistent classification results have been observed when using the imputed data. Finally, we acknowledge that this is a proof-of-concept study of using machine learning to explore refined subtype classification of obesity. In future work, further analysis using pooled data of the multi-center cohorts with random data split for training and testing/verification may further evaluate the model’s performance. The more important research that we are planning to follow up is to validate the clinical value of the identified subtypes in a prospective setting, that is, to evaluate the treatment and adverse effects of both surgical and non-surgical therapies with respect to the four obesity subtypes. This study provides feasibility data and premises to design future clinical evaluation studies.

In summary, this multi-center retrospective study identified a refined classification of obesity subtypes by mining the clinical characteristics using a machine learning approach. The four subtypes appeared to be consistent across four independent patient cohorts. This proof-of-concept study provided evidence that precise diagnosis of obesity is feasible, which has a great potential to guide therapeutic planning and decisions for different subtypes of obesity. Prospective studies are warranted to further evaluate the findings of this study.

## Data Availability Statement

The original contributions presented in the study are included in the article/**Supplementary Material**. Further inquiries can be directed to the corresponding authors.

## Ethics Statement

The studies involving human participants were reviewed and approved by The Ethical Committee of Shanghai Tenth People’s Hospital. The patients/participants provided their written informed consent to participate in this study.

## Author Contributions

SQ and SW jointly conceived the concept and supervised the study. ZL, WF, and YL performed data processing, variable selection, and data analysis. SW, DA, and LG contributed in analytical methods. XC, HY, DoZ, LD, DaZ, JY, JZ, and CM supervised, performed, and/or coordinated all data collection from the four participating hospitals and conducted data pre-processing. ZL, SW, SQ, and DA drafted the manuscript. All authors contributed to data and result interpretation. All authors contributed to the article and approved the submitted version.

## Funding

This study was supported by National Key R&D Program of China (2018YFC1314100), National Natural Science Foundation of China (81970677), Shanghai Municipality: Shanghai Outstanding Academic Leaders Plan (049), National Natural Science Foundation of China for Youth (81500687), China Scholarship Council, Shanghai Medicine and Health Development Foundation (DMRFP_I_07), Fundamental Research Funds for the Central Universities of Tongji University (22120190210). Funding sources had no involvement in study design; in the collection, analysis, or interpretation of data; in the writing of the report; or in the decision to submit the paper for publication. 

## Conflict of Interest

SW is a scientific consultant of COGNISTX, Inc. SW has a research grant funded by Amazon.

The remaining authors declare that the research was conducted in the absence of any commercial or financial relationships that could be construed as a potential conflict of interest.

## References

[1] Purcell K Sumithran P Prendergast LA Bouniu CJ Delbridge E Proietto J. The Effect of Rate of Weight Loss on Long-Term Weight Management: A Randomised Controlled Trial. Lancet Diabetes Endocrinol (2014) 2:954–62. 10.1016/S2213-8587(14)70200-1 25459211

[2] LeBlanc ES Patnode CD Webber EM Redmond N Rushkin M O’Connor EA. Behavioral and Pharmacotherapy Weight Loss Interventions to Prevent Obesity-Related Morbidity and Mortality in Adults: Updated Evidence Report and Systematic Review for the US Preventive Services Task Force. JAMA (2018) 320:1172–91. 10.1001/jama.2018.7777 PMC13151892 30326501

[3] Tobias DK Chen M Manson JE Ludwig DS Willett W Hu FB. Effect of Low-Fat Diet Interventions Versus Other Diet Interventions on Long-Term Weight Change in Adults: A Systematic Review and Meta-Analysis. Lancet Diabetes Endocrinol (2015) 3:968–79. 10.1016/S2213-8587(15)00367-8 PMC4667723 26527511

[4] Godino JG Merchant G Norman GJ Donohue MC Marshall SJ Fowler JH. Using Social and Mobile Tools for Weight Loss in Overweight and Obese Young Adults (Project SMART): A 2 Year, Parallel-Group, Randomised, Controlled Trial. Lancet Diabetes Endocrinol (2016) 4:747–55. 10.1016/S2213-8587(16)30105-X PMC5005009 27426247

[5] Lin Z Qu S. Legend of Weight Loss: A Crosstalk Between the Bariatric Surgery and the Brain. Obes Surg (2020) 30:1988–2002. 10.1007/s11695-020-04474-8 32096018

[6] Consultation WE. Appropriate Body-Mass Index for Asian Populations and its Implications for Policy and Intervention Strategies. Lancet (2004) 363:157–63. 10.1016/S0140-6736(03)15268-3 14726171

[7] Stefan N Häring H-U Hu FB Schulze MB. Metabolically Healthy Obesity: Epidemiology, Mechanisms, and Clinical Implications. Lancet Diabetes Endocrinol (2013) 1:152–62. 10.1016/S2213-8587(13)70062-7 24622321

[8] Global BMIMC Di Angelantonio E Bhupathiraju S Wormser D Gao P Kaptoge S. Body-Mass Index and All-Cause Mortality: Individual-Participant-Data Meta-Analysis of 239 Prospective Studies in Four Continents. Lancet (2016) 388:776–86. 10.1016/S0140-6736(16)30175-1 PMC4995441 27423262

[9] Expert Panel on Detection, Evaluation, and Treatment of High Blood Cholesterol in Adults. Executive Summary of The Third Report of The National Cholesterol Education Program (NCEP) Expert Panel on Detection, Evaluation, And Treatment of High Blood Cholesterol In Adults (Adult Treatment Panel III). JAMA (2001) 285:2486–97. 10.1001/jama.285.19.2486 11368702

[10] Karelis AD Rabasa-Lhoret R. Inclusion of C-Reactive Protein in the Identification of Metabolically Healthy But Obese (MHO) Individuals. Diabetes Metab (2008) 34:183–4. 10.1016/j.diabet.2007.11.004 18329310

[11] Rey-López JP de Rezende LF Pastor-Valero M Tess BH. The Prevalence of Metabolically Healthy Obesity: A Systematic Review and Critical Evaluation of the Definitions Used. Obes Rev: An Off J Int Assoc Study Obes (2014) 15:781–90. 10.1111/obr.12198 25040597

[12] Frühbeck G Kiortsis DN Catalán V. Precision Medicine: Diagnosis and Management of Obesity. Lancet Diabetes Endocrinol (2018) 6:164–6. 10.1016/S2213-8587(17)30312-1 28919063

[13] DeGregory KW Kuiper P DeSilvio T Pleuss JD Miller R Roginski JW. A Review of Machine Learning in Obesity. Obes Rev: An Off J Int Assoc Study Obes (2018) 19:668–85. 10.1111/obr.12667 PMC8176949 29426065

[14] Green M Strong M Razak F Subramanian S Relton C Bissell P. Who are the Obese? A Cluster Analysis Exploring Subgroups of the Obese. J Public Health (2016) 38:258–64. 10.1093/pubmed/fdv040 25889387

[15] Acosta A Camilleri M Shin A Vazquez-Roque MI Iturrino J Burton D. Quantitative Gastrointestinal and Psychological Traits Associated With Obesity and Response to Weight-Loss Therapy. Gastroenterology (2015) 148:537–46.e534. 10.1053/j.gastro.2014.11.020 25486131 PMC4339485

[16] Ogden LG Stroebele N Wyatt HR Catenacci VA Peters JC Stuht J. Cluster Analysis of the National Weight Control Registry to Identify Distinct Subgroups Maintaining Successful Weight Loss. Obes (Silver Spring) (2012) 20:2039–47. 10.1038/oby.2012.79 PMC4562400 22469954

[17] Heymsfield SB Wadden TA. Mechanisms, Pathophysiology, and Management of Obesity. New Engl J Med (2017) 376:254–66. 10.1056/NEJMra1514009 28099824

[18] Mechanick JI Apovian C Brethauer S Timothy Garvey W Joffe AM Kim J. Clinical Practice Guidelines for the Perioperative Nutrition, Metabolic, and Nonsurgical Support of Patients Undergoing Bariatric Procedures - 2019 Update: Cosponsored by American Association of Clinical Endocrinologists/American College of Endocrinology, The Obesity Society, American Society for Metabolic and Bariatric Surgery, Obesity Medicine Association, and American Society of Anesthesiologists. Obes (Silver Spring) (2020) 28(4). 10.1002/oby.22719 32202076

[19] Ahmad A. Dey L. A K-Mean Clustering Algorithm for Mixed Numeric and Categorical Data. Data Knowledge Engineer (2007) 63:503–27. 10.1016/j.datak.2007.03.016

[20] Bacher J Wenzig K Vogler M. SPSS TwoStep Cluster-A First Evaluation. p. 23 (2004).

[21] Jaccard P. The Distribution of the Flora in the Alpine Zone. 1. New Phytol (1912) 11:37–50. 10.1111/j.1469-8137.1912.tb05611.x

[22] Eckel N Li Y Kuxhaus O Stefan N Hu FB Schulze MB. Transition From Metabolic Healthy to Unhealthy Phenotypes and Association With Cardiovascular Disease Risk Across BMI Categories in 90 257 Women (the Nurses’ Health Study): 30 Year Follow-Up From a Prospective Cohort Study. Lancet Diabetes Endocrinol (2018) 6:714–24. 10.1016/S2213-8587(18)30137-2 29859908

[23] Tchernof A Després J-P. Pathophysiology of Human Visceral Obesity: An Update. Physiol Rev (2013) 93:359–404. 10.1152/physrev.00033.2011 23303913

[24] Neeland IJ Ross R Després J-P Matsuzawa Y Yamashita S Shai I. Visceral and Ectopic Fat, Atherosclerosis, and Cardiometabolic Disease: A Position Statement. Lancet Diabetes Endocrinol (2019) 7:715–25. 10.1016/S2213-8587(19)30084-1 31301983

[25] Álvarez-Lario B Macarrón-Vicente J. Uric Acid and Evolution. Rheumatol (Oxford) (2010) 49:2010–5. 10.1093/rheumatology/keq204 20627967

[26] Sinha S Schwartz RA. Juvenile Acanthosis Nigricans. J Am Acad Dermatol (2007) 57:502–8. 10.1016/j.jaad.2006.08.016 17592743

[27] Rosenfield RL Ehrmann DA. The Pathogenesis of Polycystic Ovary Syndrome (PCOS): The Hypothesis of PCOS as Functional Ovarian Hyperandrogenism Revisited. Endocr Rev (2016) 37:467–520. 10.1210/er.2015-1104 27459230 PMC5045492

