# Supplementary material for: Machine Learning to Identify Metabolic Subtypes of Obesity: A Multi-Center Study
Source: Front Endocrinol (Lausanne). 2021 Jul 14;12:713592. doi: 10.3389/fendo.2021.713592 (PMC8317220; doi:10.3389/fendo.2021.713592)
Supplement: Supplementary file 1 [file DataSheet_1.pdf]

Machine learning to identify metabolic subtypes of obesity: A multi-center study

Ziwei Lin, MD, PhD [1,2 †]; Wenhuan Feng, MD, PhD [3 †]; Yanjun Liu, MD, PhD [4 †]; Chiye Ma, MD, PhD [5]; Dooman Arefan, PhD [2]; Donglei Zhou, MD, PhD [1]; Xiaoyun Cheng, MD, PhD [1]; Jiahui Yu, MD [4]; Long Gao, PhD [6,2]; Lei Du, MD, PhD [1]; Hui You, MM [1]; Jiangfan Zhu, MD, PhD [1,5]; Dalong Zhu, MD, PhD [3]; Shandong Wu, PhD [2,7 *]; Shen Qu, MD, PhD [1 *]

[1] Endocrinology and Metabolism Center, National Metabolic Management Center, Division of Metabolic Surgery for Obesity and Diabetes, Shanghai Tenth People's Hospital, School of Medicine, Tongji University, Shanghai, China

[2] Department of Radiology, University of Pittsburgh, Pittsburgh, PA, USA

[3] Department of Endocrinology, Drum Tower Hospital Affiliated to Nanjing University Medical School, Nanjing, China

[4] The Center of Gastrointestinal and Minimally Invasive Surgery, Chengdu Third People's Hospital, Southwest Jiaotong University, Chengdu, China

[5] Department of Bariatric and Metabolic Surgery, Shanghai East Hospital, Tongji University, Shanghai, China

[6] College of Computer, National University of Defense Technology, Changsha, China

[7] Department of Biomedical Informatics, Department of Bioengineering, Intelligent Systems Program, University of Pittsburgh, Pittsburgh, PA, USA

[†] These authors have contributed equally to this work and share first authorship.

* These authors share senior authorship

Correspondence:

Shandong Wu

Room 322, 3240 Craft Place, Pittsburgh, PA, 15213, USA

Email: wus3@upmc.edu

Shen Qu

Shanghai Tenth People's Hospital, Tongji University School of Medicine, Shanghai 200072, P.R. China

Email：qushencn@hotmail.com

Table of Contents

Supplementary Methods **3**

Patients' inclusion and exclusion criteria 3

Measurements 3

Calculations 4

Definition of metabolic disorders 4

Reference 6

Supplementary Figures **7**

Figure S1. Distribution of patients and incidences of metabolic diseases according to traditional classification paradigms for overweight / obesity 7

Figure S2. Patient distributions in each cohort with respect to the four clusters generated from machine learning 8

Figure S3. Comparison of the three classification variables with sex stratification across the four clusters generated from Cohort-1 using k-means 9

Figure S4. Changes of metabolic features during follow-up after bariatric surgery in patients from Cohort-1B 10

Supplementary Tables **11**

Table S1. Regression models for prediction of AUC of glucose 11

Table S2. Regression models for prediction of AUC of insulin 12

Table S3. Cluster centers in Cohort-1 with twostep method 13

Table S4. Performance of assigning patients in each verification cohort to the four clusters generated by two-step on the main cohort, Cohort-1 13

Table S5. Comparison of anthropometric examination and fat distribution across the four clusters generated from Cohort-1 using k-means and normal-weight controls 14

Table S6. Comparison of organs function across the four clusters generated from Cohort-1 using k-means and normal-weight controls 15

Supplementary Methods

Patients' inclusion and exclusion criteria

For patient cohort:

Patients with overweight/obesity were included with a body mass index (BMI) ≥ 24kg/m^2 (according to the WHO criteria (1)).

Patients were excluded if they: (i) had ever undertaken a bariatric surgery before the first visit of this study; (ii) had taken exogenous insulin, medication that affects glucose metabolism, or on uric acid (UA) drugs before this study; (iii) were diagnosed with type 1 diabetes, secondary diabetes, hereditary disease, or severe disease (e.g., malignant tumor, heart failure, liver failure, etc.); (iv) were in gestation of lactation; or (v) have substantial missing clinical data related to this study.

For control cohort:

Patients with normal-weight were included with BMI between 18.5 and 24kg/m^2 (according to the WHO criteria (1)).

Patients were excluded if they: (i) had ever undertaken a bariatric surgery; (ii) had taken exogenous insulin, medication that affects glucose metabolism, or on UA drugs before this study; (iii) were diagnosed with diabetes, hyperuricemia, hereditary disease, or severe disease (e.g., malignant tumor, heart failure, liver failure, etc.); (iv) were in gestation of lactation; or (v) have substantial missing clinical data related to this study.

Measurements

In Cohort-1, a standardized health questionnaire was completed by nurses at the patient's baseline visits. The questionnaire covered the participants' basic demographic and lifestyle information, personal and family history, and medicine history.

Anthropometric measurements and clinical examination were performed at the baseline visit for both Cohort-1A and Cohort-1B, and at each follow-up visit after the bariatric surgery for Cohort-1B. Measurements were performed at 7:00-9:00 a.m. following a 10-12 hours overnight fast. All participants underwent anthropometric evaluation including height, weight, waist, hip and neck circumference, heart rate, blood pressure, and examinations for acanthosis nigricans, purple striae, and polytrichia. Then, oral glucose tolerance test (OGTT, 75g glucose) was performed with 0, 30, 60, 120, and 180 min sampling for plasma glucose and insulin.

Plasma glucose was determined using the hexokinase activity assay; total cholesterol (TC) and triglyceride (TG) were measured using oxidase peroxidase method; high density lipoprotein cholesterol (HDL-c) was measured using polyethene-glycol ether method; low density lipoprotein cholesterol (LDL-c) was measured using the peak particle diameter method; UA, alanine aminotransferase (ALT), aspartate aminotransferase (AST), γ-glutamyl transferase (γGT), and creatinine (Cr) were measured by the enzymatic method; albumin was measured by the bromocresol green method; total protein was measured using the biuret method and globulin was calculated by total protein minus albumin; total bilirubin (TBil) was measured by the diazo method; all above were performed with a Cobas c701 Analyzer (Roche Diagnostics, Mannheim, Germany). Glycosylated hemoglobin (HbA1c) was determined using high-performance liquid chromatography (HPLC) with a Tosoh HLC-723 G8 automated HPLC analyzer (Tosoh, Japan). Plasma insulin and testosterone were measured using electrochemiluminescence immunoassay with a Cobas 6000 (Roche Diagnostics, Mannheim, Germany). Free triiodothyronine (fT3), free thyroxine (fT4), and thyroid stimulating hormone (TSH) were measured using chemiluminescence immunoassay with ADVIA Centaur XP automatic chemiluminescence immunoassay system (Siemens, New York, USA). Urine albumin (UALB) was measured using pyrogallol red method with a Hitachi 7180 biochemistry autoanalyzer (Hitachi, Tokyo, Japan).

Fat distribution were examined by dual energy x-ray absorptiometry (DXA) with QDR4500W (Hologic, Waltham, USA). Ultrasound imaging was performed with Acuson Sequoia 512 (Siemens, Mountain View, CA), Acuson S2000 (Siemens, Erlangen,

Germany), or Logiq E9 (GE Healthcare, Milwaukee, Wisconsin, US). Liver steatosis was assessed by controlled attenuation parameter (CAP) and liver fibrosis was assessed by stiffness measurement with a liver transient elastography of Fibroscan 502 (Echosens, Paris, France). The cut-off points for hepatic steatosis of < 11 %, 11-34 %, 34-67 %, and ≥ 67 % were CAP < 238 dB/m, 238-259 dB/m, 259-292 dB/m, and ≥ 292 dB/m, respectively; the cut-off points for hepatic stiffness of F0-F1, F2, F2-F3, F3-F4, and F4 were measurements of < 7.3 kPa, 7.3-9.7 kPa, 9.7-12.4 kPa, 12.4-17.5 kPa, and ≥ 17.5 kPa, respectively, according to the machine.

For patients in Cohort-2, oral glucose tolerance test (OGTT, 75g glucose) was performed with 0, 30, 60, and 120 min sampling for plasma glucose and insulin measurements. Plasma glucose was determined using hexokinase activity assay with a TBA-200FR analyzer (Toshiba, Tokyo, Japan). Plasma insulin was measured using electrochemiluminescence immunoassay with a Cobas e601 (Roche Diagnostics, Mannheim, Germany). HbA1c was determined using HPLC with a Tosoh HLC-723 G8 automated HPLC analyzer (Tosoh, Japan). UA, TC, TG, Cr, ALT, AST were measured with Beckman AU5421 (Beckman, Brea, USA).

For patients in Cohort-3, OGTT (75g glucose) was performed with 0, 60, and/or 120 min sampling for plasma glucose and insulin measurements. Plasma glucose was determined using hexokinase activity assay with a BS-380 biochemical analyzer (Mindray, Shenzhen, China). Plasma insulin was measured using electrochemiluminescence immunoassay with a Beckman Coulter Unicell DXI 800 (Beckman, Fullerton, CA, USA). HbA1c was determined using HPLC with a Tosoh HLC-723 G8 automated HPLC analyzer (Tosoh, Japan). UA, TC, TG, Cr, ALT, AST were measured with Beckman AU5421 (Beckman, Brea, USA).

For patients in Cohort-4, plasma glucose was determined using hexokinase activity assay and UA was measured using enzyme colorimetry with a Cobas c701 Analyzer (Roche Diagnostics, Mannheim, Germany). Plasma insulin was measured using electrochemiluminescence immunoassay with a Cobas e411 (Roche Diagnostics, Mannheim, Germany). HbA1c was determined using HPLC with a Cobas Integra (Roche Diagnostics, Mannheim, Germany).

Patients in Cohort-0 had undergone the measurements using the same methods in the same hospital of Cohort-1. Data were not available in some anthropometrical evaluations (acanthosis nigricans, purple striae, polytrichia and circumferences), blood testing (sex hormones and thyroid hormones), urine testing and all the non-blood examinations in this normal-weight cohort.

Calculations

Excess body weight was calculated as the difference between body weight and healthy weight with BMI of 24 kg/m^2.

Pancreatic β-cell function was estimated using the insulinogenic index (IGI) (2) and homoeostasis model assessment of β-cell function (HOMA-β) (3). IGI was calculated as △insulin $_{0\text{-}30min}$ / △glucose $_{0\text{-}30min}$. HOMA-β was calculated as 20 × insulin $_{0min}$/(glucose $_{0min}$-3.5). Insulin resistance was determined by homeostatic model assessment of insulin resistance (HOMA-IR, an estimation of mainly hepatic insulin resistance) (3) as glucose $_{0min}$ × insulin $_{0min}$ /22.5. Insulin sensitivity was measured by whole-body insulin sensitivity index (WBISI, an estimation of both hepatic and peripheral tissue insulin sensitivity) (4) as 10,000/square root of ([glucose $_{0min}$ × 18 × insulin $_{0min}$] × [mean glucose × 18 × mean insulin during OGTT]), and mean glucose or insulin during OGTT were calculated as arithmetic mean of measurements at 0, 30, 60, and 120 min. Disposition indices (DI) were used to estimate relative insulin secretion compared to insulin resistance or sensitivity and calculated as HOMA-β/HOMA-IR and IGI × WBISI. For all the formulas above, glucose and insulin were calculated in mmol/l and mU/l, respectively.

Definition of metabolic disorders

Traditional BMI categories were defined using criteria according to Department of Disease Control, National Health and Family Planning Commission of China (NHFPC), and Cooperative Meta-Analysis Group of the Working Group on Obesity in China (WGOC) (5), which are the WHO criteria (1) adjusted for Chinese, as follows: (i) normal weight: BMI 18.5-24 kg/m^2; (ii)

overweight: BMI 24-28 kg/m^2; (iii) obesity: BMI ≥ 28 kg/m^2 (obese I: BMI 28-30 kg/m^2; obese II: BMI 30-35 kg/m^2; obese III: BMI ≥35 kg/m^2).

Traditional metabolic healthy/unhealthy obesity was defined according to The National Cholesterol Education Program Adult Treatment Panel III (NCEP-ATP III) (6), Chinese Diabetes Society (CDS) criteria 2004 and 2019 (which are NCEP-ATP III criteria adjusted for Chinese) (7), and Karelis criteria (8).

The NCEP-ATP III definition of metabolic abnormality required individuals to have three or more of the following components: (i) waist circumference > 102cm (men) or > 88cm (women); (ii) blood pressure ≥ 130/85 mmHg or use of antihypertensive drugs; (iii) fasting glucose ≥ 6.1 mmol/L or use of medications for diabetes; (iv) TG ≥ 1.7 mmol/L or use of lipid-lowering drugs; (v) HDL-c < 1.04 mmol/l (men), < 1.30 mmol/l (women).

CDS 2004 definition of metabolic abnormality required individuals to have three or more of the following components: (i) BMI ≥ 25kg/m^2; (ii) blood pressure ≥ 140/90 mmHg or use of antihypertensive drugs; (iii) fasting glucose ≥ 6.1 mmol/L and/or OGTT 2hr glucose ≥ 7.8 mmol/L, or use of medications for diabetes; (iv) TG ≥ 1.7 mmol/L and/or HDL-c < 0.9 mmol/L (men), < 1.0 mmol/L (women).

CDS 2019 definition of metabolic abnormality required individuals to have three or more of the following components: (i) waist circumference ≥ 90cm (men) or ≥85cm (women); (ii) blood pressure ≥ 130/85 mmHg or diagnosed hypertension and on antihypertensive therapy; (iii) fasting glucose ≥ 6.1 mmol/L and/or OGTT 2hr glucose ≥ 7.8 mmol/L, or confirmed diabetes that is under treatment; (iv) fasting TG ≥ 1.7 mmol/L; (v) fasting HDL-C < 1.04 mmol/L.

Karelis' criteria of metabolic abnormality required individuals to have two or more of the following components: (i) homeostatic model assessment of insulin resistance (HOMA-IR) ≥ 2.7; (ii) CRP ≥ 3.0 mg/l; (iii) TG > 1.7 mmol/L; (iv) LDL-c ≥2.6 mmol/L or use of lipid-lowering drugs; (v) HDL-c ≤ 1.3 mmol/L.

Hypertension was defined as systolic pressure (SP) ≥ 140 mmHg and/or diastolic pressure (DP) ≥ 90 mmHg, or confirmed hypertension that is under treatment.

Diabetes was defined according to the WHO 1999 criteria (9) as follows: fasting glucose ≥ 7.0 mmol/l and/or OGTT 2hr glucose ≥ 11.1 mmol/l, and/or confirmed diabetes that is under treatment.

Hypercholesterolemia was defined as TC ≥ 5.2 mmol/l. Hypertriglyceridemia was defined as TG ≥ 1.7 mmol/l. HDL-c < 0.9 mmol/l in men or < 1.0 mmol/l in women was defined as hypo-HDL. LDL-c ≥ 3.35 mmol/l was defined as hyper-LDL. Dyslipidemia was defined as with any of the above abnormalities, or confirmed dyslipidemia that is under treatment.

Metabolic syndrome was defined using the CDS 2004 criteria: (i) BMI ≥ 25kg/m^2; (ii) blood pressure ≥ 140/90 mmHg or use of antihypertensive drugs; (iii) fasting glucose ≥ 6.1 mmol/L and/or OGTT 2hr glucose ≥ 7.8 mmol/L, or use of medications for diabetes; (iv) TG ≥ 1.7 mmol/L and/or HDL-c < 0.9 mmol/L (men) or < 1.0 mmol/L (women). Metabolic syndrome diagnosis required individuals to have three or more of the above components.

Hyperuricemia was defined as UA ≥ 420 μmol/l for men or ≥ 360 μmol/l for women, or confirmed hyperuricemia that is under treatment.

Women hyper-testosterone was defined as testosterone ≥ 1.42 nmol/l.

Polycystic ovary syndrome (PCOS) was defined according to Rotterdam consensus workshop group (10) as follows: (i) Oligo- and/or anovulation; (ii) Clinical and/or biochemical signs of hyperandrogenism; (iii) Polycystic ovaries (PCO); PCOS diagnosis required individuals to have two or more of the above components and exclusion of other etiologies.

Men hypo-testosterone was defined as testosterone < 8.64nmol/l.

Microalbuminuria was defined as UALB ≥ 30 mg/l or albumin to creatine ratio (ACR) ≥ 30 mg/g in the early morning urine.

Nonalcoholic fatty liver disease (NAFLD) was defined by abdomen ultrasound or Fibroscan of CAP ≥ 259 dB/m (indicating liver steatosis ≥ 34%), and exclusion of other etiologies. NAFLD with increased ALT or AST was defined as NAFLD with ALT ≥ 50 U/L or AST ≥ 40 U/L.

Reference

1. WHO Expert Consultation. Appropriate body-mass index for Asian populations and its implications for policy and intervention strategies. Lancet. 2004;363(9403):157-63. Epub 2004/01/17. doi: 10.1016/S0140-6736(03)15268-3.
2. Seltzer HS, Allen EW, Herron AL, Jr., Brennan MT. Insulin secretion in response to glycemic stimulus: relation of delayed initial release to carbohydrate intolerance in mild diabetes mellitus. J Clin Invest. 1967;46(3):323-35. Epub 1967/03/01. doi: 10.1172/JCI105534.
3. Matthews DR, Hosker JP, Rudenski AS, Naylor BA, Treacher DF, Turner RC. Homeostasis model assessment: insulin resistance and beta-cell function from fasting plasma glucose and insulin concentrations in man. Diabetologia. 1985;28(7):412-9. Epub 1985/07/01. doi: 10.1007/BF00280883.
4. Matsuda M, DeFronzo RA. Insulin sensitivity indices obtained from oral glucose tolerance testing: comparison with the euglycemic insulin clamp. Diabetes Care. 1999;22(9):1462-70. doi: 10.2337/diacare.22.9.1462.
5. Zhou B, Cooperative Meta-Analysis Group of the Working Group on Obesity in China. Predictive values of body mass index and waist circumference for risk factors of certain related diseases in Chinese adults-study on optimal cut-off points of body mass index and waist circumference in Chinese adults. Biomed Environ Sci. 2002;15(1):83-96.
6. Expert Panel on Detection, Evaluation, and Treatment of High Blood Cholesterol in Adults. Executive summary of the third report of the National Cholesterol Education Program (NCEP) Expert Panel on Detection, Evaluation, and Treatment of High Blood Cholesterol in Adults (Adult Treatment Panel III). JAMA. 2001;285(19):2486-97. doi: 10.1001/jama.285.19.2486.
7. Jia W, Weng J, Zhu D, Ji L, Lu J, Zhou Z, Zou D, Guo L, Ji Q, Chen L, Chen L, Dou J, Guo X, Kuang H, Li L, Li Q, Li X, Liu J, Ran X, Shi L, Song G, Xiao X, Yang L, Zhao Z. Standards of medical care for type 2 diabetes in China 2019. Diabetes Metab Res Rev. 2019;35(6):e3158. doi: 10.1002/dmrr.3158.
8. Karelis AD, Rabasa-Lhoret R. Inclusion of C-reactive protein in the identification of metabolically healthy but obese (MHO) individuals. Diabetes Metab. 2008;34(2):183-4. doi: 10.1016/j.diabet.2007.11.004.
9. Alberti KG, Zimmet PZ. Definition, diagnosis and classification of diabetes mellitus and its complications. Part 1: diagnosis and classification of diabetes mellitus provisional report of a WHO consultation. Diabet Med. 1998;15(7):539-53. Epub 1998/08/01. doi: 10.1002/(SICI)1096-9136(199807)15:7<539::AID-DIA668>3.0.CO;2-S.
10. Rotterdam ESHRE/ASRM-Sponsored PCOS Consensus Workshop Group. Revised 2003 consensus on diagnostic criteria and long-term health risks related to polycystic ovary syndrome (PCOS). Hum Reprod. 2004;19(1):41-7. doi: 10.1016/j.fertnstert.2003.10.004.

Supplementary Figures

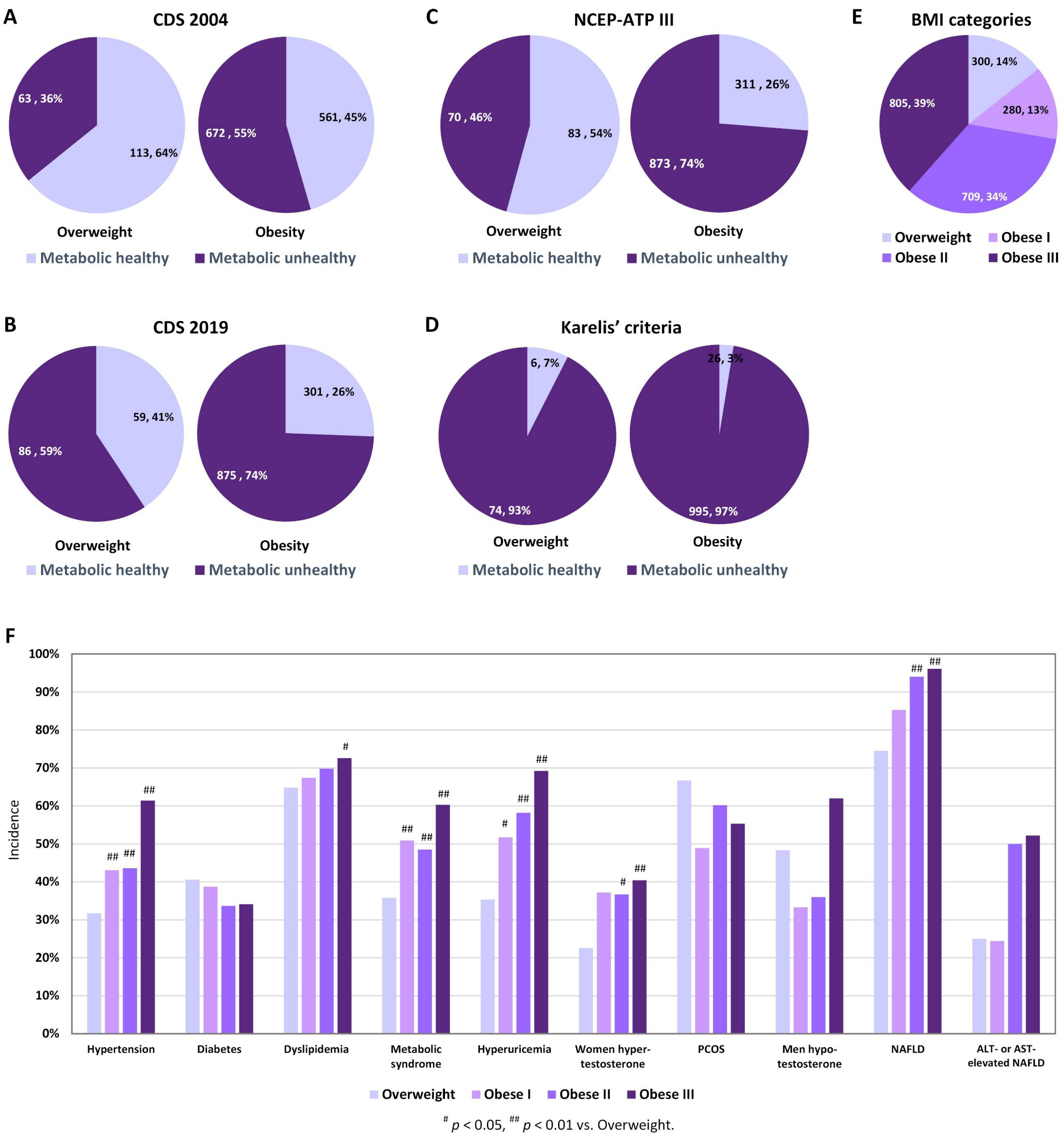

Figure S1. Distribution of patients and incidences of metabolic diseases according to traditional classification paradigms for overweight / obesity.

A-D, Distribution of patients with healthy/unhealthy obesity according to Chinese diabetes society (CDC) 2004 criteria (A), CDC 2019 criteria (B), The National Cholesterol Education Program Adult Treatment Panel III (NCEP-ATP III) criteria (C), and Karelis' criteria (D). **E,** Distribution of patients according to traditional BMI categories for overweight / obesity. Data was shown as N (patients' number) and its percentage over the full cohort. **F,** Incidences of obese comorbidities in subgroups of obesity according to traditional BMI categories. Analysis was based on patients from the full cohort of Shanghai Tenth People's Hospital (total N=2094). ALT: alanine aminotransferase; AST: aspartate aminotransferase; NAFLD: nonalcoholic fatty liver disease; PCOS: polycystic ovary syndrome.

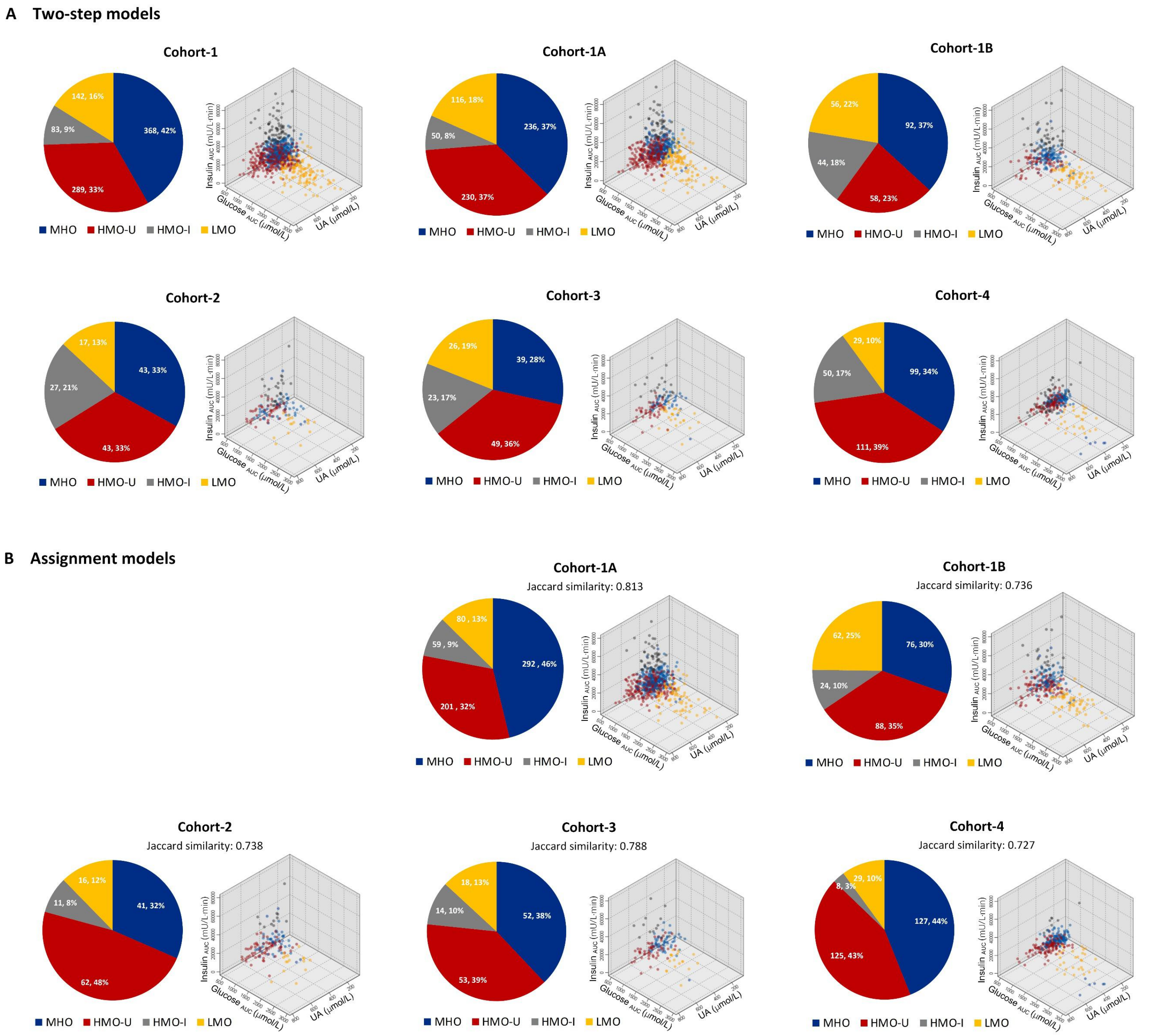

Figure S2. Patient distributions in each cohort with respect to the four clusters generated from two-step clustering.

A, Clusters generated independently from each individual cohort by using two-step clustering. **B,** Clusters generated by assigning patients in each verification cohort to the main model generated from Cohort-1 using two-step clustering. Data in the pie plots were shown as N (patient number) and its percentage over the cohort. HMO-I: hypermetabolic obesity hyperinsulinemia subtype; HMO-U: hypermetabolic obesity hyperuricemia subtype; LMO: hypometabolic obesity; MHO: metabolic healthy obesity.

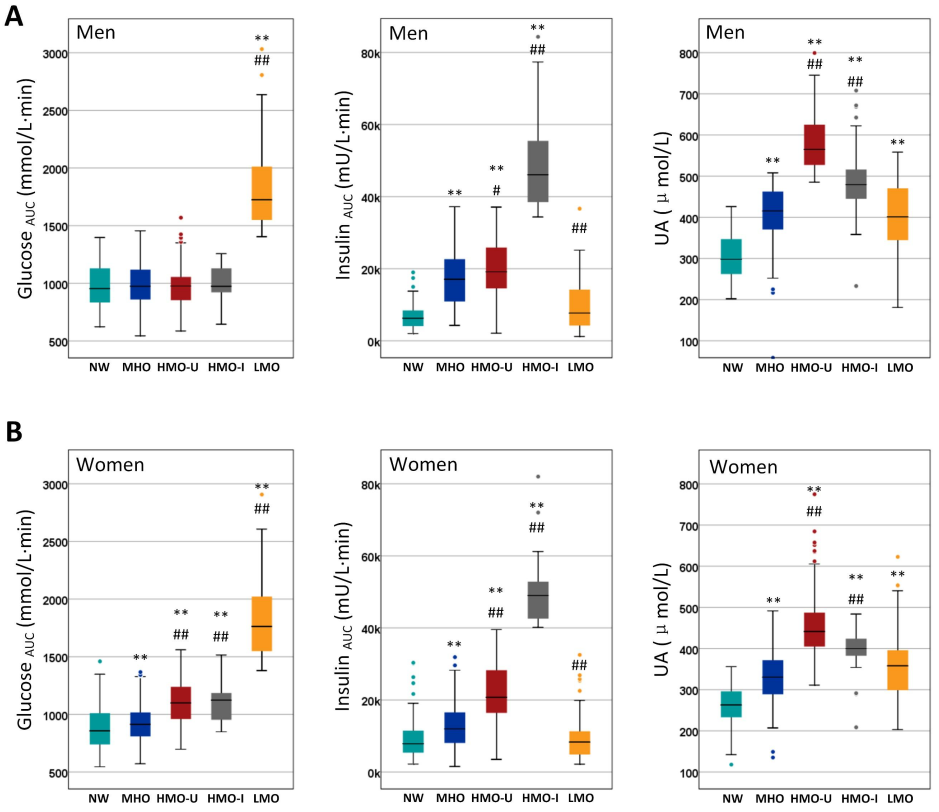

* $p < 0.05$, ** $p < 0.01$ vs. NW; # $p < 0.05$, ## $p < 0.01$ vs. MHO.

Figure S3. Comparison of the three classification variables with sex stratification across the four clusters generated from Cohort-1 using k-means.

P values after Bonferroni correction were adjusted for age and sex. HMO-I: hypermetabolic obesity hyperinsulinemia subtype; HMO-U: hypermetabolic obesity hyperuricemia subtype; LMO: hypometabolic obesity; MHO: metabolic healthy obesity; NW: normal weight control.

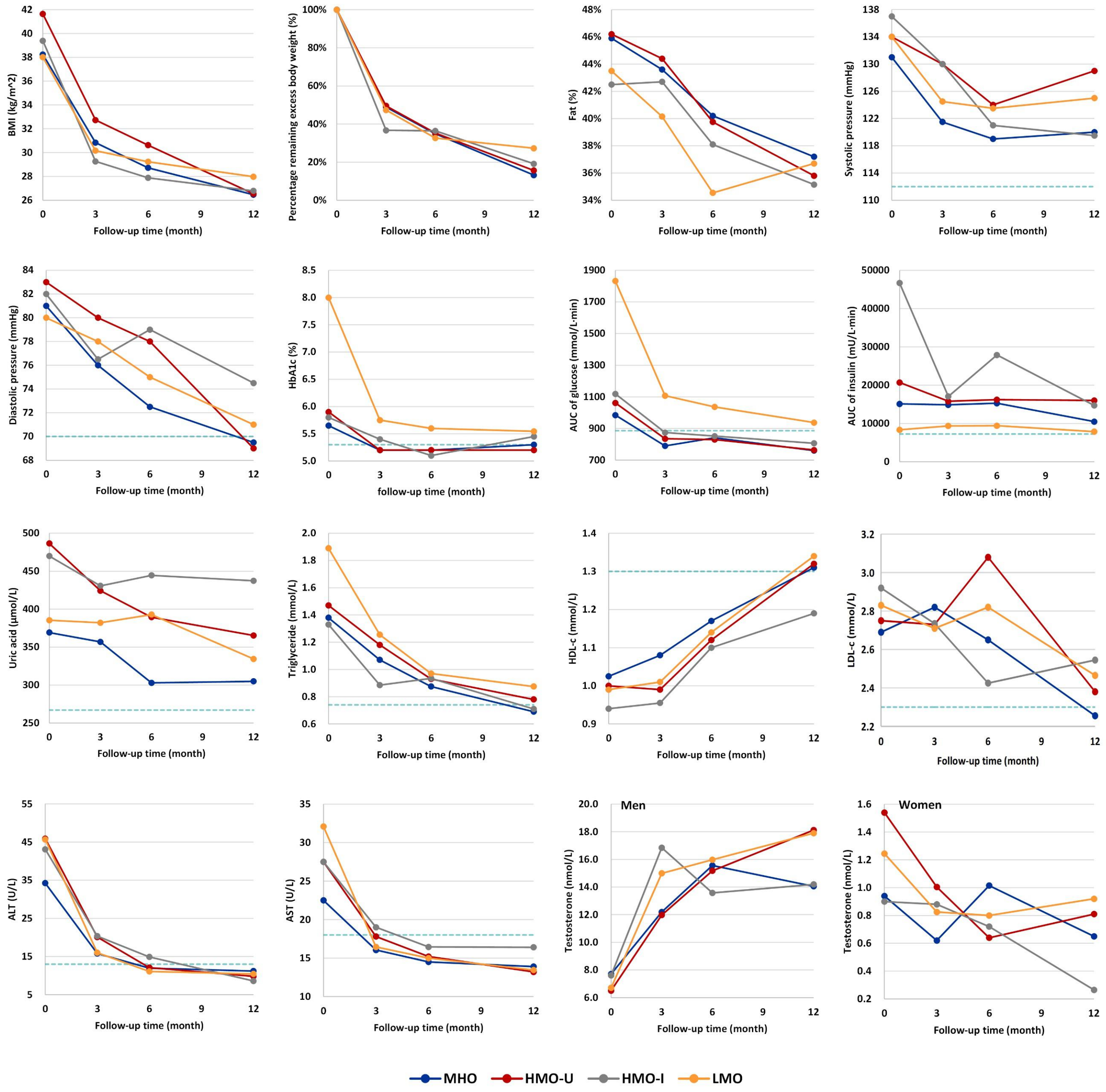

Figure S4. Changes of metabolic features during follow-up after bariatric surgery in patients from Cohort-1B.

In Cohort-1B (250 patients received bariatric surgery), 143 (57%), 116 (46%), and 98 (39%) had follow-ups at 3, 6, and 12 months, respectively. Changes of BMI, percentage of remaining excess body weight, body fat percentage, blood pressure, HbA1c, area under the curve (AUC) of glucose and of insulin, uric acid, triglyceride, high density lipoprotein cholesterol (HDL-c), low density lipoprotein cholesterol (LDL-c), alanine aminotransferase (ALT), aspartate aminotransferase (AST), and testosterone are shown at baseline and 3, 6, 12 months follow-up. Green dashed lines are the level of features in normal-weight controls at baseline (if available) which are shown as reference. HMO-I: hypermetabolic obesity hyperinsulinemia subtype; HMO-U: hypermetabolic obesity hyperuricemia subtype; LMO: hypometabolic obesity; MHO: metabolic healthy obesity.

Supplementary Tables

Table S1. Regression models for prediction of AUC of glucose.

Model	Available OGTT data	Adjusted R in training dataset	Adjusted R in testing dataset	Predictors	*β*	Standardized *β*	*t* test	*P*
Model 1	0, 60, 120 min	0.995	0.996	Constant	74.23		12.32	<0.001
				Glucose $_{60min}$	58.40	0.60	72.92	<0.001
				Glucose $_{120min}$	29.09	0.31	33.57	<0.001
				Glucose $_{0min}$	29.89	0.15	21.34	<0.001
				Insulin $_{120min}$	-0.06	-0.02	-3.69	<0.001
				Insulin $_{60min}$	0.04	0.02	2.62	0.009
Model 2	60, 120 min	0.991	0.993	Constant	146.30		22.25	<0.001
				Glucose $_{60min}$	62.82	0.65	61.63	<0.001
				Glucose $_{120min}$	36.42	0.38	34.74	<0.001
				Insulin $_{120min}$	-0.15	-0.06	-6.77	<0.001
				Insulin $_{60min}$	0.06	0.03	3.02	0.003
Model 3	60 min	0.975	0.976	Constant	153.09		13.40	<0.001
				Glucose $_{60min}$	94.73	0.96	107.05	<0.001
				Insulin $_{60min}$	-0.20	-0.08	-8.35	<0.001
Model 4	0, 120 min	0.946	0.951	Constant	181.37		10.69	<0.001
				Glucose $_{120min}$	66.13	0.70	33.86	<0.001
				Glucose $_{0min}$	59.40	0.29	14.22	<0.001
				Insulin $_{0min}$	0.46	0.03	2.26	0.024
Model 5	120 min	0.929	0.945	Constant	205.83		5.09	<0.001
				Glucose $_{120min}$	73.84	0.79	23.46	<0.001
				HbA1c	45.07	0.16	4.71	<0.001
Model 6	0 min	0.865	0.860	Constant	-110.52		-2.13	0.034
				Glucose $_{0min}$	100.83	0.54	12.46	<0.001
				HbA1c	107.34	0.37	8.52	<0.001

Models were trained with Cohort-1 patients (n=882), among which 70% of the samples was used as training dataset and 30% as testing dataset. AUC: area under the curve; OGTT: oral glucose tolerance test.

Table S2. Regression models for prediction of AUC of insulin.

Model	Available OGTT data	Adjusted R in training dataset	Adjusted R in testing dataset	Predictors	***β***	Standardized ***β***	***t* test**	***P***
Model 1	0, 60, 120 min	0.987	0.984	Constant	2676.06		7.39	<0.001
				$Insulin_{60min}$	67.43	0.75	66.99	<0.001
				$Insulin_{120min}$	27.51	0.27	26.44	<0.001
				$Glucose_{60min}$	-500.94	-0.14	-10.14	<0.001
				$Insulin_{0min}$	30.37	0.06	7.07	<0.001
				$Glucose_{0min}$	361.71	0.05	4.36	<0.001
				$Glucose_{120min}$	122.28	0.04	2.31	0.021
Model 2	60, 120 min	0.984	0.984	Constant	4124.09		12.47	<0.001
				$Insulin_{60min}$	67.92	0.75	63.68	<0.001
				$Insulin_{120min}$	27.72	0.29	25.55	<0.001
				$Glucose_{60min}$	-457.50	-0.13	-8.92	<0.001
				$Glucose_{120min}$	233.01	0.07	4.42	<0.001
Model 3	60 min	0.963	0.962	Constant	4508.01		8.92	<0.001
				$Insulin_{60min}$	86.26	0.96	87.44	<0.001
				$Glucose_{60min}$	-200.19	-0.06	-5.02	<0.001
Model 4	0, 120 min	0.897	0.837	Constant	8815.42		9.22	<0.001
				$Insulin_{120min}$	71.48	0.74	33.99	<0.001
				$Glucose_{120min}$	-980.50	-0.28	-9.48	<0.001
				$Insulin_{0min}$	135.79	0.23	11.06	<0.001
				$Glucose_{0min}$	481.43	0.06	2.11	0.036
Model 5	120 min	0.871	0.828	Constant	12215.30		16.18	<0.001
				$Insulin_{120min}$	81.25	0.84	42.47	<0.001
				$Glucose_{120min}$	-756.13	-0.21	-10.75	<0.001
Model 6	0 min	0.628	0.643	Constant	29506.31		10.13	<0.001
				$Insulin_{0min}$	267.67	0.49	11.81	<0.001
				HbA1c	-2089.30	-0.21	-3.14	0.002
				$Glucose_{0min}$	-991.56	-0.16	-2.32	0.021

Models were trained with Cohort-1 patients (n=882), among which 70% of the samples was used as training dataset and 30% as testing dataset. AUC: area under the curve; OGTT: oral glucose tolerance test.

Table S3. Cluster centers in Cohort-1 with two-step method.

	MHO	HMO-U	HMO-I	LMO
**Men**				
Glucose $_{AUC}$, mmol/L·min	943	1025	985	1707
Insulin $_{AUC}$, mU/L·min	18595	21138	54369	10357
Uric acid, µmol/L	435	611	475	385
**Women**				
Glucose $_{AUC}$, mmol/L·min	906	1121	1068	1910
Insulin $_{AUC}$, mU/L·min	13638	17463	44525	8478
Uric acid, µmol/L	317	442	410	351

HMO-U: hypermetabolic obesity hyperuricemia subtype; HMO-I: hypermetabolic obesity hyperinsulinemia subtype; LMO: hypometabolic obesity; MHO: metabolic healthy obesity; AUC: area under the curve during oral glucose tolerance test.

Table S4. Performance of assigning patients in each verification cohort to the four clusters generated by two-step clustering on the main cohort, Cohort-1.

	Cohort-1A			Cohort-1B			Cohort-2			Cohort-3			Cohort-4			Mean		
	ACC	SEN	SPE	ACC	SEN	SPE	ACC	SEN	SPE	ACC	SEN	SPE	ACC	SEN	SPE	ACC	SEN	SPE
MHO	0.892	0.975	0.843	0.792	0.630	0.886	0.785	0.651	0.851	0.861	0.923	0.837	0.855	0.929	0.816	0.837	0.822	0.847
HMO-U	0.834	0.709	0.905	0.800	0.828	0.792	0.838	0.977	0.770	0.854	0.837	0.864	0.792	0.793	0.792	0.824	0.829	0.825
HMO-I	0.986	1.000	0.985	0.920	0.545	1.000	0.877	0.407	1.000	0.934	0.609	1.000	0.855	0.160	1.000	0.914	0.544	0.997
LMO	0.915	0.612	0.983	0.960	0.964	0.959	0.977	0.882	0.991	0.927	0.654	0.991	0.979	0.897	0.988	0.952	0.802	0.982
**Mean**	0.907	0.824	0.929	0.868	0.742	0.909	0.869	0.729	0.903	0.894	0.756	0.923	0.870	0.695	0.899	0.882	0.749	0.913

Patients assigned to model were based on log-likelihood distance. ACC: Accuracy; HMO-I: hypermetabolic obesity hyperinsulinemia subtype; HMO-U: hypermetabolic obesity hyperuricemia subtype; LMO: hypometabolic obesity; MHO: metabolic healthy obesity; SEN: Sensitivity; SPE: Specificity.

Table S5. Comparison of anthropometric examination and fat distribution across the four clusters generated from Cohort-1 using k-means and normal-weight controls.

	NW	MHO	HMO-U	HMO-I	LMO
**Anthropometric examination**					
Systolic pressure, mmHg	112 (106, 121)	131 (120, 143) **	134 (124, 146) **##	138 (128, 144) **	138 (79, 152) **##
Diastolic pressure, mmHg	70 (64, 77)	83 (75, 90) **	86 (77, 94) **##	84 (78, 90) **	86 (80, 95) **
SP - DP, mmHg	43 (38, 49)	48 (40, 58) **	48 (39, 58) **	50 (44, 56) **	51 (81, 64) **##
Heart rate, bpm	81 (74, 89)	81 (73, 92)	84 (79, 94) **#	88 (80, 97) **#	83 (78, 94) **##
Height, m	1.63 (1.59, 1.68)	1.67 (1.62, 1.76) **	1.68 (1.62, 1.77) **#	1.75 (1.69, 1.80) **#	1.69 (1.62, 1.75) **
Weight, kg	57.5 (52.0, 62.3)	95.7 (82.0, 109.2) **	105.0 (91.3, 123.0) **##	115.8 (94.9, 129.2) **##	100.6 (88.3, 119.2) **
Excess body weight, kg	NA	27.6 (17.4, 38.9)	35.6 (24.9, 50.7) ##	42.3 (25.4, 50.3) ##	32.5 (20.8, 47.1)
Neck circumference, cm	NA	40 (37, 44)	42 (39, 45) ##	44 (41, 46) ##	43 (40, 46) ##
Waist circumference, cm	NA	107 (98, 118)	113 (105, 123) ##	117 (106, 126) ##	115 (104, 124) ##
Hip circumference, cm	NA	113 (107, 120)	118 (110, 127) ##	118 (110, 126)	113 (107, 123)
Patients with purple striae, %	NA	23.4%	23.6%	23.0%	17.4%
Patients with polytrichia, %	NA	4.5%	8.1%	10.4%	8.2%
**Fat distribution (DXA)**					
Fat, %	NA	43.5 (38.5, 47.2)	44.1 (40.8, 47.4)	41.5 (36.4, 45.4)	42.5 (37.3, 46.2)
Fat mass index, kg/m^2	NA	15.54 (12.67, 17.71)	16.67 (14.60, 19.61) ##	14.42 (13.10, 17.81)	15.00 (12.96, 18.12)
Fat free mass index, kg/m^2	NA	19.84 (17.72, 22.83)	21.48 (19.13, 23.83) ##	22.55 (20.57, 24.16)	21.51 (18.73, 23.41)
Android / gynoid fat percentage ratio	NA	1.19 (1.12, 1.30)	1.17 (1.10, 1.26)	1.27 (1.21, 1.39)	1.27 (1.22, 1.36)

P values after Bonferroni correction are adjusted for age and sex. DP: diastolic pressure; DXA: dual energy x-ray absorptiometry; HMO-I: hypermetabolic obesity hyperinsulinemia subtype; HMO-U: hypermetabolic obesity hyperuricemia subtype; LMO: hypometabolic obesity; MHO: metabolic healthy obesity; NA: not available; NW: normal weight control; SP: systolic pressure. * P<0.05, ** P<0.01 vs. NW; # P<0.05, ## P<0.01 vs. MHO.

Table S6. Comparison of organs function across the four clusters generated from Cohort-1 using k-means and normal-weight controls.

	NW	MHO	HMO-U	HMO-I	LMO
**Liver function**					
ALT (U/L)	13.0 (10.0, 16.0)	32.8 (20.8, 62.1) **	47.7 (29.2, 87.4) **##	56.8 (30.4, 83.8) **##	55.1 (29.8, 95.6) **##
AST (U/L)	18.0 (15.0, 22.0)	23.1 (17.9, 33.8) **	30.6 (21.0, 49.0) **##	30.4 (22.0, 51.6) **	32.5 (21.7, 58.4) **##
γGT, U/L	14.0 (11.0, 20.0)	33.6 (21.7, 54.0) **	43.8 (29.3, 69.6) **##	33.7 (24.9, 46.5) **	46.5 (31.5, 71.5) **##
Albumin, g/L	47 (45, 48)	44 (41, 46) **	44 (42, 46) **	43 (42, 47) **	43 (40, 45) **
Globulin, g/L	27 (26, 29)	28 (25, 30) **	30 (27, 32) **##	27 (25, 30)	28 (25, 32)
Total bilirubin, μmol/L	11.0 (8.7, 14.3)	9.0 (6.1, 12.0) **	10.1 (7.2, 14.0)	8.4 (5.8, 12.3) **	10.1 (8.2, 14.2) ##
Patients with <11%, 11-34%, 34-67%, ≥67% hepatic steatosis (transient elastography), respectively, %	NA	2.0%, 5.0%, 11.0%, 82.0%	1.0%, 3.9%, 2.9%, 92.2%	0.0%, 0.0%, 4.3%, 95.7%	1.9%, 0.0%, 1.9%, 96.2%
Patients with liver stiffness degree of F0-F1, F2, F2-F3, F4 (transient elastography), respectively, %	NA	51.0%, 20.0%, 14.0%, 8.0%, 7.0%	50.5%, 15.5%, 14.6%, 11.7%, 7.8%	30.4%, 30.4%, 13.0%, 4.3%, 21.7% #	40.4%, 19.2%, 21.2%, 11.5%, 7.7%
**Kidney function**					
Urine microalbumin, mg/L	NA	19.0 (12.8, 32.1)	29.3 (15.4, 82.8) #	19.6 (10.1, 25.9)	30.7 (13.1, 89.5)
ACR, mg/g	NA	16.0 (10.1, 25.7)	21.7 (9.6, 53.9)	13.8 (8.4, 17.2)	33.3 (14.6, 70.0)
**Thyroid**					
fT3 (pmol/L)	NA	5.17 (4.79, 5.61)	5.19 (4.73, 5.62)	5.40 (4.84, 6.04)	4.99 (4.49, 5.44)
fT4 (pmol/L)	NA	15.57 (14.00, 17.48)	16.00 (14.53, 17.41)	16.15 (14.93, 17.01)	16.83 (15.12, 18.75) ##
TSH (mU/L)	NA	2.19 (1.51, 3.20)	2.53 (1.63, 3.43)	2.28 (1.63, 3.04)	2.04 (1.46, 2.90)
Patients with thyroid nodule (ultrasound), %	NA	40.0%	34.7%	60.0%	37.5%
Patients with thyroid diffuse disease (ultrasound), %	NA	29.1%	13.9% #	40.0%	29.2%
**Gonad**					
Testosterone (men), nmol/L	NA	9.40 (5.45, 12.15)	8.11 (5.40, 11.30)	7.51 (4.18, 10.26)	7.50 (5.09, 10.40)
Female patients with uterine myoma (ultrasound), %	NA	12.9%	14.1%	11.1%	35% #

P values after Bonferroni correction are adjusted for age and sex. ALT: alanine aminotransferase; AST: aspartate aminotransferase; ACR: albumin creatinine ratio; fT3: Free triiodothyronine; fT4: free thyroxine; HMO-I: hypermetabolic obesity hyperinsulinemia subtype; HMO-U: hypermetabolic obesity hyperuricemia subtype; LMO: hypometabolic obesity; MHO: metabolic healthy obesity; NA: not available; NW: normal weight control; TSH: thyroid stimulating hormone; γGT: γ-glutamyl transferase. * P<0.05, ** P<0.01 vs. NW; # P<0.05, ## P<0.01 vs. MHO.
